# Age-related determinants of geriatric depression: an analysis of theory-driven and data-driven approaches

**DOI:** 10.1186/s12913-026-14760-3

**Published:** 2026-05-22

**Authors:** Fatih Orhan

**Affiliations:** Gülhane Vocational School of Health, University of Health Sciences, Ankara, Turkey

**Keywords:** Geriatric depression, Aging, Logistic regression, Machine learning, Age groups, Risk factors, Functional independence, GDS-30, TEPA-2023

## Abstract

**Background:**

Geriatric depression is a prevalent mental health condition whose risk profile may vary across age strata. This study examined whether depression risk and protective factors differ across age groups (65–74, 75–84, 85+) among community-dwelling older adults in Turkey.

**Methods:**

Data were derived from the Turkey Elderly Profile Survey 2023 (TEPA-2023), a nationally representative multi-stage cluster sample. Among 29,785 individuals surveyed, the final analytical sample comprised 8,370 adults aged 65 years and older. Depression was defined using the Geriatric Depression Scale-30 (GDS-30) with a cut-off score of ≥ 11. In the theory-driven phase, binary logistic regression models including 24 independent variables were estimated for the overall sample and for separate age groups, with model validation based on stratified 10-fold cross-validation. In the data-driven phase, multiple machine learning algorithms were compared using accuracy, recall, F1-score, and area under the ROC curve (AUC).

**Results:**

The prevalence of depression increased significantly with age, from 39.9% in the 65–74 group to 53.4% in the 75–84 group and 67.7% in the 85 + group (χ² = 242.40, *p* < 0.001). In the overall logistic regression model, the strongest risk factor was unhappiness (OR = 2.516), whereas the strongest protective factor was functional independence as measured by Lawton-Brody IADL (OR = 0.386). The overall model showed good discrimination (AUC = 0.812; McFadden R² = 0.245). Age-stratified analyses showed that the number of significant predictors decreased with advancing age (13→9→5), and obesity showed a protective effect only in the 85 + group (OR = 0.506, *p* = 0.016). In the data-driven phase, ten machine learning algorithms were compared using stratified 10-fold cross-validation. Gradient Boosting achieved the highest overall AUC (0.813), while SVM-RBF yielded the best discrimination in the 75–84 (0.806) and 85+ (0.828) groups. Permutation importance analysis converged with logistic regression findings, consistently ranking unhappiness, self-rated health, Lawton-Brody IADL, and physical activity frequency as top predictors. Across age strata, predictor prominence shifted from education and income in the Under-75 group toward BMI and disability indicators in the 85 + group.

**Conclusions:**

The risk profile of geriatric depression changes substantially with advancing age. While socioeconomic factors appear more prominent in younger-old adults, health status and functional independence become more dominant in later old age. The combined use of theory-driven regression and data-driven machine learning provides a more comprehensive framework for identifying age-related depression patterns in older adults.

## Introduction

Depression has emerged as one of the leading causes of disease burden attributable to mental disorders over the past 30 years and is particularly prevalent among the elderly population [1, 2]. Global studies indicate that the lifetime prevalence of depression is 7.5% in men and 13.6% in women, with rates increasing to 20.1% in men and 34.0% in women by the age of 75. This suggests that more than one in five individuals may experience depressive symptoms at least once in their lifetime [3, 4]. Geriatric depression is a widespread and multidimensional issue that not only reduces quality of life [5] but also adversely affects both social and physical functioning in older adults [6, 7].

The existing literature emphasizes that depression results from complex interactions between biological, psychological, socioeconomic, and environmental factors [8, 9]. However, the extent to which these factors vary with age remains underexplored. The notion that different age groups—65–74 years, 75–84 years, and ≥ 85 years—may have distinct risk factors and protective elements represents a crucial dimension in the study of geriatric depression. Traditional research has predominantly relied either on theory-driven approaches that formulate hypotheses based on theoretical frameworks before conducting data analysis [10, 11] or on data-driven approaches that apply large-scale dataset analyses [12, 13]. However, integrating these two approaches may provide a more comprehensive understanding of the underlying mechanisms of depression and allow for the development of age-specific clinical interventions.

In line with gerontological literature, this study stratified age into 65–74 (young-old), 75–84 (middle-old), and ≥ 85 years (oldest-old). Such stratification has been widely applied in epidemiological and mental health research to reflect clinically meaningful differences in functional decline, comorbidity burden, and psychosocial vulnerabilities across older populations [14–16]. For example, studies have reported distinct patterns of depression prevalence and associated risk factors across these age strata. While age is inherently a continuous variable, the use of categorical cut-offs in this context allows for clearer subgroup comparisons and aligns with established conventions in aging research.

In this study, we aim to combine the advantages of both approaches to examine the effects of various factors on geriatric depression, both separately for different age groups and in a unified sample for comparison. The rationale for this research is to identify age-related differences that have not yet been sufficiently addressed in the literature and to support these distinctions with concrete data that can be translated into clinical practice.

Data-driven techniques facilitate the discovery of complex patterns and predictive variables without being constrained by predefined hypotheses by leveraging large datasets and advanced algorithms, such as machine learning, natural language processing, neural networks, and factor analysis [17, 18]. Across health sciences and related fields, these methods have been applied to identify disease subtypes, predict clinical outcomes, and explore behavioral and organizational constructs [19–24]. At the same time, theory-driven approaches remain essential to ensure interpretability, conceptual coherence, and clinical or practical relevance, as shown in research on psychological, nutritional, and organizational domains [21–23, 25–27].

Taken together, evidence from prior studies demonstrates that data-driven methods can generate novel hypotheses and detect complex associations, while theory-driven frameworks provide the explanatory basis for validation and clinical translation [21, 25–30]. Their integration has been highlighted across disciplines including neurology, psychiatry, nutrition, and social sciences [21, 28–30]. However, this combined application has rarely been extended to geriatric depression, where capturing age-specific risk and protective factors is particularly important. In this study, we address this gap by combining both approaches to analyze the multidimensional factors of geriatric depression across different age groups. This integrative framework is expected to yield age-sensitive insights that may inform more effective clinical interventions and public health strategies.

This study aims to systematically examine the differences emerging across age groups by integrating theory-driven and data-driven approaches to analyze the multidimensional factors underlying geriatric depression. This integrative framework is of critical importance in developing more effective intervention strategies and enabling policymakers to devise holistic solutions to improve the mental health of the elderly population. The findings provide critical insights for health management and policymakers in developing age-appropriate intervention programs and service planning for elderly care.

Based on the literature and the study’s theoretical framework, the following hypotheses are proposed:

- H₁: Depression prevalence increases significantly across age groups (65–74 < 75–84 < 85+).
- H₂: Functional independence (Lawton-Brody IADL and Katz ADL) is a significant protective factor against geriatric depression across all age groups.
- H₃: The risk/protective factor profiles differ across age groups, with socioeconomic factors (education, income) more prominent in the young-old and health/functional factors dominating in the oldest-old.
- H₄: Theory-driven logistic regression and data-driven machine learning approaches yield concordant variable importance rankings.

## Method

### Research model and dataset

In this study, risk factors for depression among individuals aged 65 years and older were examined using the microdata from the Turkey Elderly Profile Survey 2023 (TYP-2023). The research employed a cross-sectional design and was conducted using an analytical epidemiological approach. The original dataset consists of 11,657 observations.

The dependent variable, Depression_Binary, was constructed based on the total score of the Geriatric Depression Scale (GDS-30). The operationalization of this variable is described in Sect. “Dependent variable”.

### Data collection process and sample size

The Turkey Elderly Profile Survey was conducted within the framework of a protocol signed between the Turkish Statistical Institute (TÜİK) and the Ministry of Family and Social Services. Field implementation was carried out nationwide between 23 October and 18 December 2023. The sampling frame of the study was constructed based on the Address-Based Population Registration System (ABPRS), and updated address information from the National Address Database (UAVT) was used.

The sampling method was multi-stage cluster sampling. In the first stage, clusters consisting of approximately 100 household addresses containing at least one individual aged 50 years or older, according to the ABPRS, were selected using a probability proportional to size (PPS) sampling method. Within this framework, 2,264 clusters were randomly selected. In the second stage, 10 household addresses from each selected cluster were included in the sample using systematic random sampling. The total sample size therefore consisted of 22,640 household addresses. This design ensured that the sample was probability-based and representative.

The geographical scope of the survey covered all settlements within the borders of the Republic of Türkiye. The institutional population (e.g., nursing homes, dormitories, prisons, military barracks) was excluded from the scope. However, all households living outside institutional settings and containing at least one individual aged 50 years or older were included. The estimation framework was designed to produce results both at the national level and at the NUTS-1 (Statistical Region Units Classification Level 1) level, consisting of 12 regions. Therefore, the dataset has representative power at both national and regional levels.

Within the selected 22,640 households, data were collected from 29,785 individuals aged 50 years and older. Among these individuals, 11,657 were aged 65 years and above. The planning of the sample size to enable estimates both for the overall population of Türkiye and for the NUTS-1 level increases the statistical power and generalizability of the study.

Regarding the response rate, during the weighting process conducted by TÜİK, design weights were calculated, non-response adjustments were applied, and integrative calibration and trimming procedures were performed to generate the final weighting factor. This process was intended to reduce potential non-response bias and strengthen the representativeness of the sample. Although non-response adjustment and calibration procedures are described in the methodology, a specific response rate percentage is not reported. Since this information has not been publicly disclosed by TÜİK, we are unable to provide a precise response rate.

Prior to field implementation, households were sent a “Household Information Letter” explaining the purpose, scope, and principles of data usage of the survey. This practice was implemented to both increase participation rates and improve data quality. The fieldwork was conducted under the coordination of TÜİK’s 26 Regional Directorates.

The data collection method followed a mixed-mode approach. The Household Members’ Basic Characteristics Form and the Household Questionnaire were completed with the household respondent, while the Individual Questionnaire was administered directly to individuals aged 50 years and older. The primary data collection method was Computer-Assisted Personal Interviewing (CAPI), where interviewers recorded responses directly into the system using portable computers. In cases where face-to-face contact was not possible, Computer-Assisted Telephone Interviewing (CATI) was applied. For individuals unable to respond due to advanced age or health problems, an appropriate proxy respondent was used; however, perceptual or subjective questions were not asked through proxies. This approach represents a methodological precaution aimed at reducing measurement error and bias.

In conclusion, TYPA 2023 is a methodologically robust official statistical survey based on probability-based multi-stage cluster sampling, representative at both national and regional levels, incorporating non-response adjustments and calibration procedures, supported by pre-survey informational letters, and utilizing both CAPI and CATI data collection methods. These characteristics strengthen both the internal validity and external validity of the research findings.

The adequacy of the sample size for logistic regression analysis was evaluated based on the Events Per Variable (EPV) criterion proposed by Peduzzi et al. [31]. In the overall model, the total sample size is 10,348, with a depression prevalence of 46.7%, and the model includes 24 independent variables, excluding the intercept. Based on the least frequent category, the calculated EPV value is 201.4. This value corresponds to approximately 20 times the recommended minimum threshold of EPV ≥ 10 and clearly exceeds the more stringent EPV ≥ 20 criterion. According to the Peduzzi formula, the minimum required sample size is 514, whereas the current sample is approximately 20 times larger than this requirement. Therefore, for the overall model, the risk of overfitting appears to be low, and the coefficient estimates can be considered statistically reliable.

In the age-stratified analyses, sample adequacy is largely maintained. The EPV values were calculated as 130.5 for the 65–74 age group and 57.2 for the 75–84 age group, both of which comfortably meet the recommended thresholds. In contrast, the 85 + age group yielded an EPV value of 9.0, which falls slightly below the classical EPV ≥ 10 criterion. This indicates that estimates for the oldest age group should be interpreted with greater caution. Nevertheless, considering that the methodological literature acknowledges a degree of flexibility, suggesting that EPV values in the 5–10 range may still be acceptable, the sample structure of this group can be regarded as borderline but acceptable rather than clearly insufficient. Accordingly, the findings related to the 85 + group should be discussed within the methodological limitations section.

The overall model fit was evaluated using the Likelihood Ratio test, which demonstrated that the full model provides a significant improvement over the null model (LR χ² = 3,361.95; df = 24; *p* < 0.001). The McFadden Pseudo R² value was 0.2351, corresponding to a Cohen’s f² = 0.307, which falls within the medium-to-large effect size range. This level indicates that the model possesses not only statistical significance but also meaningful explanatory power in practical and clinical terms. Furthermore, post-hoc power analyses conducted at the variable level showed that the statistical power for the majority of significant predictors was well above 80%, with an average power of 94.6%. These findings suggest that the main results of the model carry a low risk of Type II error.

Finally, minimum detectable effect size analyses indicate that, given the current sample size, even small changes in odds ratios can be statistically detected. In the overall sample, with 80% statistical power and α = 0.05, even relatively small increases in risk such as OR ≈ 1.057 are detectable. In subgroup analyses, this threshold increases with age, meaning that larger effect sizes are required for detection in older groups, particularly in the 85 + category. Overall, the sample size can be considered highly sufficient for the overall model and the two larger age subgroups, while for the oldest age group it is borderline but methodologically defensible. Taken together, this structure indicates that the main findings of the study rest on a statistically robust foundation in terms of statistical power.

### Missing data analysis and sample

When examining the missing data pattern in the dataset, it was found that 1,309 observations (11.2%) had missing values simultaneously in five variables (monthly income, happiness level, GDS-30 total score, GDS-30 category, and the dependent variable DEPRESYON_BINARY). This complete overlap suggests that these participants either did not respond to the relevant sections of the questionnaire or that subjective/perceptual questions were not administered to respondents interviewed through a proxy.

**Fig. 1:**
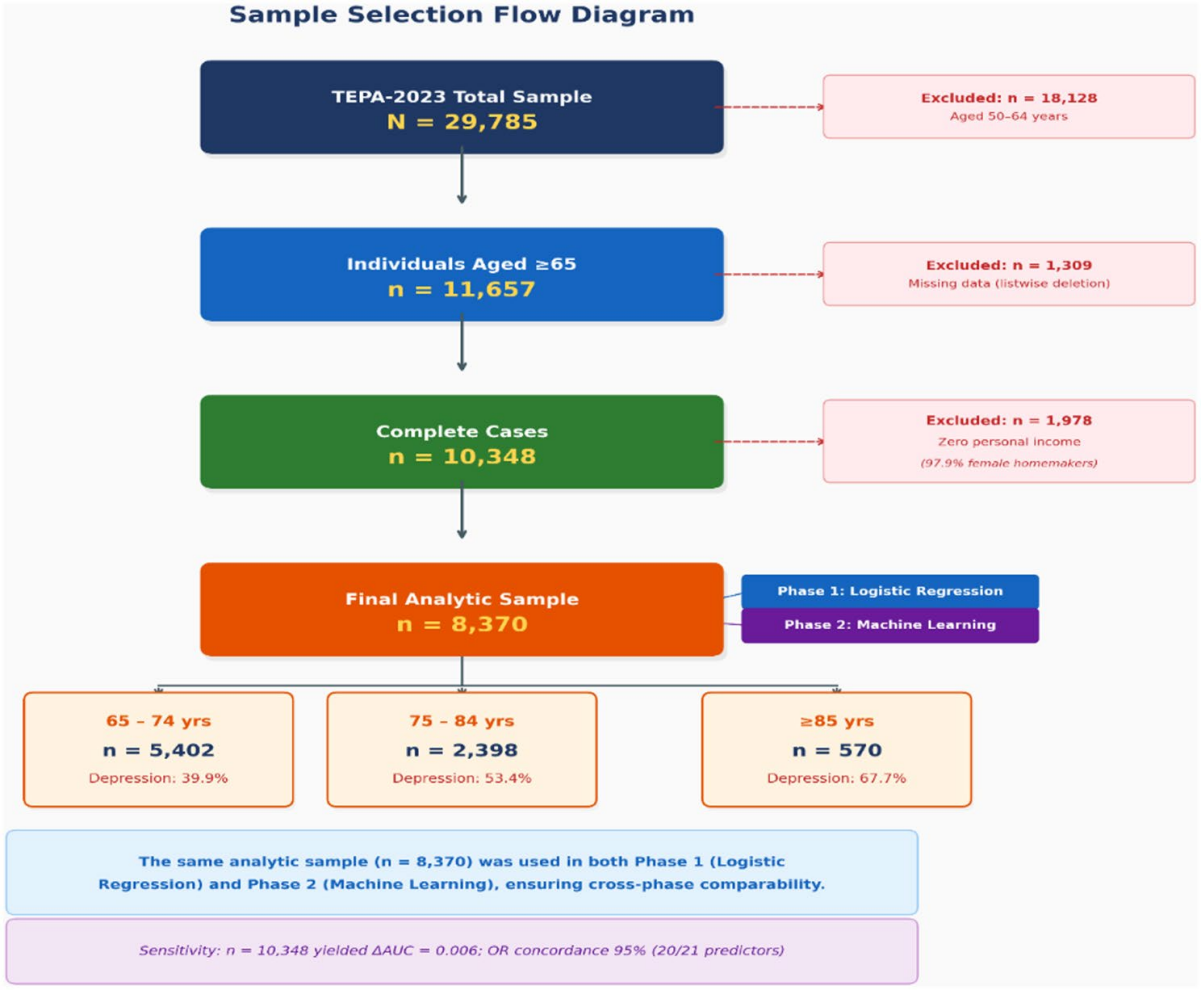
Sample selection flow diagram

Across the 24 independent variables used in the analysis, a total of 3,927 cells out of 279,768 contained missing values, corresponding to an overall missing data rate of 1.40%. Since the missing values were concentrated within a single group of observations, the listwise deletion method was applied. Accordingly, the 1,309 observations with missing data were excluded, resulting in a reduced sample of *n* = 10,348 (Fig. 1).

In the second stage, a decision was made to exclude 1,978 participants (19.1%) who reported their personal monthly income as 0 TL. This decision was based on the systematic demographic pattern observed in this group. Among participants reporting income = 0 TL, 97.9% (*n* = 1,936) were women, 95.9% (*n* = 1,897) were married, and 100% were living with another person. This profile strongly indicates housewives who do not have an individual income but are financially dependent on their spouse’s household income. In Türkiye, particularly in rural areas and older age groups, a substantial proportion of women are not formally employed, and therefore appear as having 0 TL individual income in survey data. However, this situation reflects household income distribution and gender roles rather than actual poverty.

For the income variable to function as a meaningful predictor in the logistic regression model, it must represent genuine socioeconomic differences. If participants reporting income = 0 TL were included, the income variable would largely measure an interaction between employment status and gender rather than personal income level, thereby substantially reducing the interpretability of the variable. Additionally, including this group would reduce the proportion of women in the sample from 54.6% to 44.3%, significantly altering the gender distribution.

Following these exclusions, the final analytical sample size was determined as *n* = 8,370. The same sample was used for both Phase 1 (logistic regression) and Phase 2 (machine learning) analyses, ensuring direct comparability between the results of the two phases. The sample was stratified into three age groups: 65–74 years (*n* = 5,402), 75–84 years (*n* = 2,398), and 85 years and older (*n* = 570).

To test whether the exclusion decision affected the results, all analyses were repeated using the expanded sample (*n* = 10,348) that included participants reporting income = 0 TL. The sensitivity analysis results indicate that model performance remained largely unchanged: the AUC difference was only 0.006 (0.812 vs. 0.806), the accuracy difference was 0.010, and 20 of the 21 predictor variables (95.2%) showed effects in the same direction (risk or protective) in both samples. These findings confirm that the exclusion of participants with income = 0 TL did not alter the main results of the study and that the sample selection decision is robust.

### Definition and transformation of variables

#### Dependent variable

Depression status (DEPRESYON_BINARY) was coded as a binary categorical variable. Individuals with a GDS-30 total score of 11 or higher were classified as depressed (1), while those scoring below 11 were classified as non-depressed (0).

#### Independent variables and coding

A total of 24 independent variables were included in the analysis. These variables were grouped into four categories: demographic, health-related, functional, and lifestyle variables. The transformations applied are described below:

Age variable: Instead of using the continuous age variable, three categorical age groups were created: 65–74, 75–84, and 85 + years. The 65–74 age group was defined as the reference category. This transformation was implemented to address the high multicollinearity problem (VIF > 50) that emerged when the continuous age variable was included in the model. Additionally, this categorization enables subgroup analyses to compare age-specific risk profiles. The decision to code the age variable categorically as 65–74, 75–84, and 85+, rather than as a continuous variable, is based on both theoretical and methodological considerations. The gerontology literature conceptualizes old age not as a homogeneous and linear continuum, but rather as a life stage composed of qualitatively distinct phases. The distinction between the young-old and the old-old, introduced by Neugarten [32], was further expanded by Baltes and Smith [33] through the conceptualization of the third age and fourth age. In particular, the fourth age is emphasized as being categorically different from earlier stages in terms of biological frailty, functional dependency, and psychosocial loss. Similarly, empirical evidence suggests that as age increases, health, functional capacity, and well-being do not change linearly but exhibit threshold-like discontinuities [34]. This framework supports modeling age as a stage-based structure rather than a linear covariate. The 65–74 / 75–84 / 85 + classification used in this study is also consistent with international standards, thereby enhancing comparability across studies.

From a methodological perspective, the use of a continuous variable in logistic regression requires the assumption of a linear relationship between the predictor and the log-odds of the outcome [35]. This assumption was examined for the age variable using the grouped logit approach, and the age–logit relationship was found to deviate from strict linearity. In particular, a plateau trend was observed in the 75–84 age range, accompanied by minor declines in some sub-intervals, followed by marked increases in the oldest ages. This fluctuating pattern indicates that a linear functional form cannot fully capture the relationship between age and depression. Because misspecification of the functional form in regression modeling may lead to biased estimates [36], categorical coding allows the relationship to be captured without imposing a linear structure, enabling separate parameter estimation for each age group.

From a clinical and policy perspective, the categorical approach is also more practical. Although the annual odds ratio obtained from the continuous model (OR = 0.990) may be statistically significant, it is not intuitively meaningful in clinical terms and does not produce a clear threshold that can be directly used for health planning. In contrast, age-group-based estimates present risk differences in a concrete and actionable manner. Since geriatric screening and intervention programs are typically designed according to age categories, this modeling approach is more meaningful at the implementation level. Furthermore, the primary research question of this study is to examine whether depression predictors differ across age groups. Addressing this objective requires treating age not merely as a control variable but as a stratification variable. If age were modeled as a continuous variable, it would be necessary to include numerous age × predictor interaction terms, substantially increasing model complexity. In contrast, stratified analysis, by estimating separate models for each age group, demonstrated that the number of significant predictors decreases substantially in advanced ages, clearly revealing age-specific risk patterns.

Finally, the potential concern that categorical coding may result in information loss was empirically tested. The discriminative performance of the continuous and categorical models is nearly identical (AUC = 0.8139 for the continuous model; AUC = 0.8140 for the categorical model). This finding indicates that categorical modeling does not weaken predictive power and that the results are robust. The continuous model may still be reported as an additional sensitivity analysis; however, when theoretical consistency, functional form validity, clinical interpretability, and the nature of the research question are considered together, analyzing age in a categorical and stratified manner represents the more appropriate theoretical and methodological choice.

##### Income variable

Monthly individual income was rescaled by dividing the original value in Turkish Lira by **1**,**000**, resulting in the variable **Income_1000TL** (Mean = 8.08; SD = 8.58). This transformation addresses the issue that, in the original scale, the regression coefficient (beta ≈ 0.000) and the odds ratio (OR ≈ 1.000) were too small to be meaningfully interpreted. After rescaling, the OR values reflect the change in depression risk per 1,000 TL increase in income.

Functional scales: The total scores of the Lawton–Brody Instrumental Activities of Daily Living (IADL) and Katz Basic Activities of Daily Living (ADL) were transformed to the 0–1 range using min–max normalization. For both scales, higher values indicate greater independence.

Categorical variables: Binary variables (sex, living alone, chronic disease, disability report, alcohol use, WG disability) were coded as 0/1. Nominal variables were included in the model using dummy coding: the reference category for marital status was “Married”, and the reference category for BMI was “Normal BMI (18.5–24.99)”. Ordinal variables (education level, health status, tobacco use, activity level, frequency of physical activity, and happiness) were coded using sequential integer values while preserving their original ordinal structure.

Physical activity was assessed using two separate self-report items included in the TEPA-2023 survey. The General Activity Level measures the habitual intensity of an individual’s physical activity during a typical day. Participants were asked the question: “When you think about a typical day, which of the following best describes your situation?” Responses were categorized into three levels: (1) mostly sitting or standing (2), walking or moderate physical work, and (3) heavy work or physically demanding activity. This variable reflects the overall activity pattern encompassing occupational activities, household tasks, transportation, and other forms of daily mobility.

The Frequency of Physical Activity was measured using the question: “How often do you engage in physical activity, exercise, or sports?” This variable captures the frequency of intentional and structured exercise behavior. The response categories were (1) never (2), rarely (3), 1–3 times per month (4), at least once per week, and (5) every day or almost every day.

Both variables were coded as ordinal variables representing increasing levels of physical activity and were included in the regression models as ordered independent variables. Although these variables were not derived from a validated multi-item scale, they were analyzed to capture two distinct dimensions of physical activity: overall daily activity intensity and the frequency of planned exercise.

### Variable coding table and reference categories

To ensure compliance with the assumptions of logistic regression analysis, systematic coding and transformation procedures were applied to the dataset. First, binary categorical variables were converted into 0/1 format to facilitate analytical interpretation and improve model stability. Within this framework, the sex variable was recoded as male = 0 and female = 1. Similarly, the variables living alone, presence of chronic disease, disability report, and alcohol use were transformed into binary form as yes = 1 and no = 0. This coding scheme enables regression coefficients to be directly interpreted relative to the reference category.

Continuous variables and functional status indicators measured as total scores were subjected to Min–Max normalization within the 0–1 range in order to prevent numerical imbalances arising from scale differences within the regression model. In this context, the variables LAWTON_BRODY_TOTAL (range 0–8) and KATZ_TOTAL (range 0–6) were converted into proportional values, allowing them to become directly comparable within the model.

For ordinal variables, particular attention was paid to preserving the ordered structure of the categories. The education level variable, which originally contained a scattered coding structure, was reorganized into sequential ordered categories ranging from 1 to 9 to ensure analytical consistency. Similarly, the physical activity frequency variable was recoded into an ordinal scale ranging from 1 to 5. Other variables—such as self-rated health status, happiness, difficulty in daily activities, tobacco use, and activity level—were already structured as ordinal variables, and therefore their existing coding schemes were retained.

For nominal variables with multiple categories, dummy coding was applied after determining appropriate reference categories. For the marital status variable, the reference category was defined as “married”, and the remaining categories were represented by three separate dummy variables; the “unknown” category was treated as missing (NaN). Similarly, for BMI categories, “normal BMI” was selected as the reference category, and three dummy variables were created. For the GDS-30 category variable, “no depression” was used as the reference category, and two dummy variables were defined.

As a result of these transformations, the dataset was structured in a manner that is appropriate for logistic regression analysis, ensuring interpretability, statistical consistency, and methodological robustness (Table 1).

**Table 1:** Variable coding table and reference categories

Variable	Measurement Type	Coding / Value Range	Interpretation Direction
Depression_Binary (Dependent)	Binary	0 = No depression (Ref.), 1 = Depression present	GDS-30 ≥ 11
Sex	Binary	0 = Male (Ref.), 1 = Female	–
Age_75–84	Dummy	0 = No, 1 = Yes | Reference: 65–74 years	–
Age_85+	Dummy	0 = No, 1 = Yes | Reference: 65–74 years	–
Education Level	Ordinal	1 = No schooling → 9 = Doctorate	Increasing = higher education
Living Alone	Binary	0 = No (Ref.), 1 = Yes	–
Income_1000TL	Continuous	Monthly income / 1000 TL	Per 1000 TL increase
Self-Rated Health Status	Ordinal	1 = Very good → 5 = Very poor	Increasing = worse health
Chronic Disease	Binary	0 = No (Ref.), 1 = Yes	–
Disability Report	Binary	0 = No (Ref.), 1 = Yes	–
Alcohol Use	Binary	0 = No (Ref.), 1 = Yes	–
Night Sleep Duration	Continuous	Hours (0–15)	–
Tobacco Use	Ordinal	1 = Daily → 4 = Never used	Increasing = less use
Activity Level	Ordinal	1 = Mostly sedentary → 3 = Heavy physical work	Increasing = more active
Physical Activity Frequency	Ordinal	1 = Never → 5 = Every day	Increasing = more frequent
Happiness	Ordinal	1 = Very happy → 5 = Very unhappy	Increasing = more unhappiness
WG Disability	Binary	0 = No (Ref.), 1 = Yes	–
Lawton–Brody IADL Score	Continuous	0–1 (Min–Max), higher = more independent	Increasing = protective
Katz ADL Score	Continuous	0–1 (Min–Max), higher = more independent	Increasing = protective
Never Married	Dummy	0 = No, 1 = Yes | Reference: Married	–
Divorced	Dummy	0 = No, 1 = Yes | Reference: Married	–
Widowed	Dummy	0 = No, 1 = Yes | Reference: Married	–
Underweight	Dummy	0 = No, 1 = Yes | Reference: Normal BMI	–
Overweight	Dummy	0 = No, 1 = Yes | Reference: Normal BMI	–
Obese	Dummy	0 = No, 1 = Yes | Reference: Normal BMI	–

### Statistical analysis

#### Descriptive statistics

To determine the differences between depressed and non-depressed groups, the independent samples t-test was applied for continuous and ordinal variables, while the Pearson chi-square test was used for categorical variables.

#### Multicollinearity assessment

Multicollinearity among the independent variables was evaluated using the Variance Inflation Factor (VIF). A threshold value of VIF > 5 was considered indicative of potential multicollinearity. All variables were found to have VIF values below this threshold. The VIF values for all variables are presented below (Table 2):

**Table 2:** VIF analysis values

Variable	VIF	Status	Interpretation
Sex	1.936	✓ Normal	Acceptable (< 5)
Age_75–84	1.246	✓ Normal	Acceptable (< 5)
Age_85+	1.349	✓ Normal	Acceptable (< 5)
Education Level	1.410	✓ Normal	Acceptable (< 5)
Living Alone	1.721	✓ Normal	Acceptable (< 5)
Income_1000TL	1.244	✓ Normal	Acceptable (< 5)
Self-Rated Health Status	1.677	✓ Normal	Acceptable (< 5)
Chronic Disease	1.293	✓ Normal	Acceptable (< 5)
Disability Report	1.110	✓ Normal	Acceptable (< 5)
Alcohol Use	1.200	✓ Normal	Acceptable (< 5)
Night Sleep Duration	1.031	✓ Normal	Acceptable (< 5)
Tobacco Use	1.279	✓ Normal	Acceptable (< 5)
Activity Level	1.314	✓ Normal	Acceptable (< 5)
Physical Activity Frequency	1.223	✓ Normal	Acceptable (< 5)
Happiness	1.152	✓ Normal	Acceptable (< 5)
WG Disability	1.471	✓ Normal	Acceptable (< 5)
Lawton–Brody IADL Score	2.231	✓ Normal	Acceptable (< 5)
Katz ADL Score	1.735	✓ Normal	Acceptable (< 5)
Never Married	1.073	✓ Normal	Acceptable (< 5)
Divorced	1.204	✓ Normal	Acceptable (< 5)
Widowed	2.553	✓ Normal	Acceptable (< 5)
Underweight	1.037	✓ Normal	Acceptable (< 5)
Overweight	1.397	✓ Normal	Acceptable (< 5)
Obese	1.467	✓ Normal	Acceptable (< 5)

Maximum VIF: 2.553 | Threshold: 5.0 | Conclusion: No multicollinearity problem detected

#### Logistic regression analysis

Because the dependent variable has a binary categorical structure, multivariate binary logistic regression analysis was applied. The Enter method (forced entry) was used in the analyses, and all independent variables were included in the model simultaneously. For each independent variable, the regression coefficient (B), standard error (SE), Wald z statistic, p-value, odds ratio (OR), and 95% confidence interval (CI) were reported. Model fit was evaluated using the McFadden Pseudo R², Akaike Information Criterion (AIC), and the Hosmer–Lemeshow goodness-of-fit test.

The analyses were conducted at two levels:

1. a general model including all age groups, and.
2. subgroup models conducted separately for the 65–74, 75–84, and 85 + age groups.

In the subgroup analyses, the age dummy variables were excluded from the model.

#### Model validation

To evaluate the generalizability of model performance, Stratified 10-Fold Cross-Validation was applied instead of a single training/test split (80/20 split). In this method, the dataset was divided into 10 equal folds; each fold was used once as the test set, while the remaining nine folds were used as the training set. Before each fold, z-score standardization was applied to the independent variables using StandardScaler. In each fold, the following performance metrics were calculated: accuracy, precision, sensitivity (recall), F1-score, and the area under the ROC curve (AUC). The statistical significance level was set at *p* < 0.05 for all analyses.

To interpret model predictions, two complementary approaches were applied: feature importance in tree-based models and feature coefficients in logistic regression. Feature importance reflects the contribution of each variable to the model’s predictive performance and is derived from impurity reduction in decision trees [37]. Logistic regression coefficients, by contrast, quantify the direct log-odds relationship between predictors and the target variable [38–40]. While feature importance provides a relative ranking of predictors, it does not indicate direction of effect, whereas logistic regression coefficients convey both magnitude and direction of association [41].

Feature importance is most applicable in ensemble methods such as Random Forest and AdaBoost, which are well-suited to capture non-linearity and interactions, while feature coefficients are inherent to logistic regression, which assumes linearity between predictors and the outcome [42, 43]. Employing both techniques ensured a comprehensive and interpretable evaluation of key predictors.

#### Evaluation of class balance

The class distribution of the dependent variable was examined in the final analytical sample (*n* = 8,370): depression present (GDS-30 ≥ 11): *n* = 3,822 (45.7%) and no depression: *n* = 4,548 (54.3%).

The minority-to-majority class ratio was 1.19:1, which is below the commonly accepted balanced class threshold of 1.5:1 in the machine learning literature [44, 45]. The proportion of the minority class in the total sample (45.7%) is also well above the 20–30% threshold at which balancing techniques are typically recommended. Therefore, balancing methods such as SMOTE (Synthetic Minority Over-sampling Technique), random undersampling, or class_weight adjustments were not considered necessary for the general model.

Class balance was also evaluated separately for each age group. In the 65–74 age group (*n* = 5,402), the depression rate was 39.9%, corresponding to an imbalance ratio of 1.51:1, which is at the threshold level. In the 75–84 age group (*n* = 2,398), the depression rate was 53.4%, indicating an almost perfectly balanced distribution (1.15:1). In the 85 + age group (*n* = 570), the depression rate was 67.7%, corresponding to an imbalance ratio of 2.10:1; however, because the imbalance favors the depressed class, it does not negatively affect the model’s ability to identify depression cases. In all three age groups, the minority class proportion remains above 30%, indicating that no severe class imbalance is present.

To verify whether balancing techniques would alter the results, the logistic regression model was re-estimated using the class_weight = “balanced” parameter and compared with the Stratified 10-Fold Cross-Validation results. The findings were nearly identical to the standard model without balancing: the AUC remained unchanged at 0.812 in both conditions; accuracy decreased slightly from 0.747 to 0.743, and MCC decreased from 0.489 to 0.482 (a negligible difference). While sensitivity increased from 0.654 to 0.701, specificity decreased from 0.825 to 0.779. The fact that AUC and MCC remained unchanged indicates that class weighting only shifts the sensitivity–specificity balance without affecting the overall discriminative power of the model. This finding empirically confirms that the current class distribution does not require balancing procedures (Table 3).

**Table 3:** Evidence of class balance

Criterion	Value	Evaluation
Overall class ratio	1.19:1	Balanced (< 1.5:1 threshold)
Minority class proportion	45.7%	Balanced (> 30% threshold)
Age 65–74 ratio	1.51:1	Borderline (slight imbalance)
Age 75–84 ratio	1.15:1	Balanced
Age 85 + ratio	2.10:1	Slight imbalance (in favor of depression)
AUC (standard vs. balanced)	0.812 vs. 0.812	No difference
MCC (standard vs. balanced)	0.489 vs. 0.482	Negligible difference
Is balancing required?	NO	Empirical + theoretical evidence

Additionally, the Stratified K-Fold Cross-Validation method itself provides an implicit balancing mechanism by preserving class proportions within each fold [46]. The Matthews Correlation Coefficient (MCC), which is considered a metric robust to class imbalance, was also reported. In the overall model, MCC = 0.489, which is above the commonly accepted “acceptable” threshold (> 0.3) [47]. Reporting the MCC further confirms that the model demonstrates balanced performance across both classes.

#### Rationale for using a predictive model in a cross-sectional study and the limitation of causality

This study does not aim to establish a causal–explanatory model; rather, it adopts a diagnostic prediction framework. In the statistical modeling literature, it is well recognized that descriptive, explanatory, and predictive objectives are epistemologically distinct [48]. The primary objective of this study is to determine which sociodemographic, lifestyle, and functional characteristics best discriminate the current presence of geriatric depression, and thereby contribute to the development of screening strategies. Accordingly, the modeling approach aims not to test causal mechanisms, but to generate probability estimates that distinguish the presence of depression. In diagnostic prediction models, the goal is to calculate the probability of an existing condition based on variables measured simultaneously, and in this context a cross-sectional design is methodologically appropriate [49, 50]. Therefore, throughout this article, the terms “predictor,” “risk factor,” and “protective factor” do not imply causal direction; rather, they refer to variables that show statistical associations with depression status within the analytical sample.

The most important limitation of a cross-sectional design is the inability to draw causal inferences. All findings reported in this study reflect concurrent associations, and the temporal precedence between variables cannot be determined. In particular, variables such as happiness and self-rated health perception may partially overlap with depressive symptomatology, and bidirectional relationships may exist. Therefore, the reported odds ratios should not be interpreted as causal effect sizes, but rather as indicators of the strength and direction of concurrent associations. The models developed in this study do not aim to provide etiological explanations; instead, they serve as analytical tools for screening and risk stratification by identifying clusters of characteristics that co-occur with depression. Testing causal mechanisms would require longitudinal or quasi-experimental research designs.

Nevertheless, predictive modeling conducted within a cross-sectional framework can provide important analytical contributions. In the first stage, theory-driven logistic regression analysis was used to evaluate independent associations. In the second stage, machine learning classifiers were employed to test the individual-level discriminative accuracy of these variables. This two-stage approach demonstrates whether theoretical associations translate into practical classification performance. Moreover, analyses conducted across different age groups revealed that the number of predictors decreases progressively from younger-old groups to the oldest-old group, generating testable hypotheses regarding the age-related architecture of depression. Although this finding does not constitute causal evidence, it provides an analytical framework for future research.

In conclusion, this study adopts a diagnostic prediction approach within a cross-sectional design, makes no causal claims, and restricts all interpretations to the level of statistical associations. The model outputs contribute to the identification of current depression risk for screening and prioritization purposes, while the explanation of causal mechanisms is left to future longitudinal research [48–51].

## Results

Table 4 presents a comparative overview of the sociodemographic and clinical characteristics of 8,370 individuals aged 65 years and older included in the study, according to depression status. In the study sample, the prevalence of depressive symptoms was 45.7% (*n* = 3,822), highlighting the widespread nature of depression among the elderly population.

The majority of participants were in the 65–74 age group (64.5%), followed by the 75–84 age group (28.6%) and the 85 + age group (6.8%). The prevalence of depression increased markedly with age: 39.9% in the 65–74 group, 53.4% in the 75–84 group, and 67.7% in the 85 + group. This finding indicates that advanced age is a significant risk factor for depression (χ² = 242.40, *p* < 0.001).

The sample consisted of 55.7% women and 44.3% men. Interestingly, 55.6% of individuals with depressive symptoms were men, whereas 65.1% of individuals without depressive symptoms were women. This paradoxical finding suggests that depression rates were higher among men than among women in this elderly sample (χ² = 360.56, *p* < 0.001). This result contrasts with the commonly reported higher prevalence of depression among women in the general population and indicates that depression in older men may represent a particularly important public health concern.

Education level differed significantly between the depression groups (t = − 22.71, *p* < 0.001). Individuals with depressive symptoms had a significantly lower average education level (2.00 ± 1.39) compared with those without depressive symptoms (2.82 ± 1.84). Similarly, monthly income levels differed significantly between the groups; individuals with depressive symptoms had a lower average income (8.22 ± 6.28 thousand TL) compared with those without depressive symptoms (11.46 ± 9.72 thousand TL) (t = − 17.73, *p* < 0.001). These findings support the notion that low socioeconomic status is a risk factor for depression in older age.

Significant differences were observed between the groups in terms of marital status (χ² = 389.70, *p* < 0.001). Among individuals with depressive symptoms, 48.2% were widowed, compared with 27.7% among those without depressive symptoms. The proportion of individuals living alone was also significantly higher in the depression group (30.3%) compared with the non-depression group (21.3%) (χ² = 89.07, *p* < 0.001). These findings indicate that social isolation and widowhood increase the risk of depression in later life.

Self-rated health status showed clear differences between the groups (t = 36.02, *p* < 0.001). Individuals with depressive symptoms reported poorer health perceptions (3.36 ± 0.77) compared with those without depressive symptoms (2.78 ± 0.71). The presence of chronic disease was significantly higher among individuals with depressive symptoms (85.7%) compared with those without depression (70.5%) (χ² = 275.64, *p* < 0.001). Similarly, the prevalence of disability reports was markedly higher in the depression group (12.5% vs. 4.5%).

Indicators of functional capacity showed dramatic differences between the groups. The Lawton–Brody Instrumental Activities of Daily Living (IADL) score was significantly lower in individuals with depressive symptoms (0.63 ± 0.32) compared with those without depressive symptoms (0.83 ± 0.22) (t = − 33.91, *p* < 0.001). Likewise, the Katz Basic Activities of Daily Living (ADL) score was lower in the depression group (0.86 ± 0.27 vs. 0.97 ± 0.11; t = − 26.47, *p* < 0.001). The Washington Group disability indicator was present in 56.4% of individuals with depressive symptoms, compared with 23.3% of those without depression (χ² = 959.09, *p* < 0.001). These findings demonstrate that loss of functional independence is strongly associated with depression.

Night sleep duration was significantly shorter among individuals with depressive symptoms (6.74 ± 1.75 h) compared with those without depressive symptoms (7.08 ± 1.41 h) (t = − 10.06, *p* < 0.001). Physical activity frequency also differed between groups; individuals with depressive symptoms reported lower levels of physical activity (1.85 ± 1.20 vs. 2.46 ± 1.34; t = − 21.78, *p* < 0.001). Interestingly, alcohol consumption was more common among individuals without depressive symptoms (17.8%) compared with those with depressive symptoms (10.6%), suggesting that moderate alcohol consumption may potentially have a protective association in older adults. As expected, happiness levels were significantly lower among individuals with depressive symptoms (2.70 ± 0.83) compared with those without depressive symptoms (2.15 ± 0.60) (t = 34.61, *p* < 0.001).

Significant differences were also observed across BMI categories (χ² = 49.62, *p* < 0.001). The prevalence of underweight status was higher among individuals with depressive symptoms (1.9%) compared with those without depressive symptoms (0.9%). Obesity prevalence was also higher in the depression group (28.0%) compared with the non-depression group (23.2%). In contrast, the proportion of overweight individuals was higher in the non-depression group (44.9% vs. 39.4%), suggesting that being slightly overweight in older age may have a protective association.

Overall, this descriptive analysis reveals a high prevalence of depressive symptoms (45.7%) among individuals aged 65 years and older, along with significant associations with multiple sociodemographic, clinical, and lifestyle factors. All examined variables demonstrated statistically significant differences according to depression status. In particular, advanced age, male sex, lower education and income levels, widowhood, living alone, presence of chronic disease, poorer perceived health, loss of functional capacity, and lower levels of physical activity emerged as factors strongly associated with depressive symptoms. These findings should be taken into consideration in the planning of depression screening and prevention programs for older adults.

**Table 4:** Sociodemographic and clinical characteristics of participants (*n* = 8,370)

Variable	Total *n* = 8,370 n (%)	Depression Present *n* = 3,822 (45.7%) n (%)	No Depression *n* = 4,548 (54.3%) n (%)	Test Statistic	*p*-value
**Age Group**,** n (%)**				χ² = 242.40	< 0.001
65–74	5,402 (64.5)	2,155 (56.4)	3,247 (71.4)		
75–84	2,398 (28.6)	1,281 (33.5)	1,117 (24.6)		
85+	570 (6.8)	386 (10.1)	184 (4.0)		
**Sex**,** n (%)**				χ² = 360.56	< 0.001
Female	4,661 (55.7)	1,698 (44.4)	2,963 (65.1)		
Male	3,709 (44.3)	2,124 (55.6)	1,585 (34.9)		
**Education Level**,** Mean ± SD**	2.45 ± 1.70	2.00 ± 1.39	2.82 ± 1.84	t = − 22.70	< 0.001
**Marital Status**,** n (%)**				χ² = 389.70	< 0.001
Married	4,862 (58.1)	1,792 (46.9)	3,070 (67.5)		
Widowed	3,102 (37.1)	1,844 (48.2)	1,258 (27.7)		
Divorced	294 (3.5)	137 (3.6)	157 (3.5)		
Never married	112 (1.3)	49 (1.3)	63 (1.4)		
**Living Alone**,** n (%)**				χ² = 89.07	< 0.001
Yes	2,127 (25.4)	1,159 (30.3)	968 (21.3)		
**Income (1000 TL)**,** Mean ± SD**	9.98 ± 8.48	8.22 ± 6.28	11.46 ± 9.72	t = − 17.73	< 0.001
**BMI Category**,** n (%)**				χ² = 49.62	< 0.001
Underweight	110 (1.3)	71 (1.9)	39 (0.9)		
Normal	2,585 (30.9)	1,177 (30.8)	1,408 (31.0)		
Overweight	3,548 (42.4)	1,504 (39.4)	2,044 (44.9)		
Obese	2,127 (25.4)	1,070 (28.0)	1,057 (23.2)		
**Chronic Disease**,** n (%)**				χ² = 275.64	< 0.001
Present	6,483 (77.5)	3,277 (85.7)	3,206 (70.5)		
**Disability Report**,** n (%)**				χ² = 176.25	< 0.001
Present	683 (8.2)	478 (12.5)	205 (4.5)		
**Self-Rated Health Status**,** Mean ± SD**	3.04 ± 0.79	3.36 ± 0.77	2.78 ± 0.71	t = 36.02	< 0.001
**WG Disability**,** n (%)**				χ² = 959.09	< 0.001
Present	3,217 (38.4)	2,156 (56.4)	1,061 (23.3)		
**Lawton–Brody IADL**,** Mean ± SD**	0.74 ± 0.29	0.63 ± 0.32	0.83 ± 0.22	t = − 33.91	< 0.001
**Katz ADL**,** Mean ± SD**	0.92 ± 0.20	0.86 ± 0.27	0.97 ± 0.11	t = − 26.47	< 0.001
**Night Sleep (hours)**,** Mean ± SD**	6.93 ± 1.58	6.74 ± 1.75	7.08 ± 1.41	t = − 10.06	< 0.001
**Physical Activity Frequency**,** Mean ± SD**	2.18 ± 1.31	1.85 ± 1.20	2.46 ± 1.34	t = − 21.78	< 0.001
**Alcohol Use**,** n (%)**				χ² = 86.50	< 0.001
Yes	1,215 (14.5)	405 (10.6)	810 (17.8)		
**Happiness Level**,** Mean ± SD**	2.40 ± 0.76	2.70 ± 0.83	2.15 ± 0.60	t = 34.61	< 0.001

### Phase 1: Findings from theory-driven analysis

#### Prevalence of depression and its distribution across age groups

In the total sample of 8,370 individuals, the overall prevalence of depression was 45.7%. However, a marked increasing trend was observed across age groups: the prevalence rose from 39.9% among individuals aged 65–74 years to 53.4% in the 75–84 age group, and further to 67.7% among those aged 85 years and older. Correspondingly, the proportion of individuals without depression decreased with advancing age. The chi-square analysis (χ² = 242.399; *p* ≤ 0.001) indicated a statistically significant association between age and depression status. These findings suggest that advanced age is a strong risk determinant for depression and highlight the need for targeted mental health interventions, particularly for populations aged 75 years and older (Table 5).

**Table 5:** Prevalence of depression by age groups

Age	*n*	Depression Present (*n*)	Depression Present (%)	Depression Absent (*n*)	Depression Absent (%)
65–74	5402	2155	%39.9	3247	%60.1
75–84	2398	1281	%53.4	1117	%46.6
85+	570	386	%67.7	184	%32.3
TOTAL	**8370**	**3822**	**%45.7**	**4548**	**%54.3**

χ² = 242.399, *p* < 0.001 | Ratio of participants with and without depression: 0.840

#### Descriptive statistics

The independent samples *t*-test results for continuous and ordinal variables indicate that individuals with depression exhibit a more disadvantaged socioeconomic and behavioral profile. In the depression group, educational attainment and income levels were significantly lower (*p* < 0.001), whereas perceived health status was poorer. Both the level and frequency of physical activity were significantly lower among individuals with depression, suggesting that a sedentary lifestyle may co-occur with depressive symptoms. Nighttime sleep duration was also lower in the depression group. Higher levels of tobacco use and loneliness scores among depressed individuals further support the prominent role of psychosocial risk factors. In addition, lower total scores on the Lawton–Brody Instrumental Activities of Daily Living Scale and the Katz Activities of Daily Living Scale in the depression group indicate that functional capacity and quality of life are inversely associated with depression. The fact that all differences were significant at the *p* < 0.001 level demonstrates the strong statistical basis of these findings (Table 6).

**Table 6:** Continuous/ordinal variables (*t*-test)

Variable	Depression Present Mean ± SD	Depression Absent Mean ± SD	t-value	*p*	Sig
Education Status	1.999 ± 1.391	2.821 ± 1.837	-22.705	< 0.001	***
Income (1000 TL)	8.223 ± 6.278	11.463 ± 9.723	-17.729	< 0.001	***
Health Status	3.361 ± 0.773	2.777 ± 0.710	36.018	< 0.001	***
Night Sleep Duration	6.737 ± 1.747	7.085 ± 1.413	-10.060	< 0.001	***
Tobacco Use	3.388 ± 0.994	3.206 ± 1.057	8.076	< 0.001	***
Activity Status	1.295 ± 0.469	1.562 ± 0.522	-24.422	< 0.001	***
Frequency of Physical Activity	1.847 ± 1.202	2.457 ± 1.337	-21.783	< 0.001	***
Happiness	2.695 ± 0.833	2.152 ± 0.598	34.610	< 0.001	***
Lawton–Brody Total Score	0.630 ± 0.321	0.830 ± 0.216	-33.910	< 0.001	***
Katz Total Score	0.859 ± 0.265	0.973 ± 0.107	-26.471	< 0.001	***

Similarly, chi-square analyses of categorical variables revealed strong associations. The prevalence of depression was significantly higher among women, individuals living alone, those with chronic illnesses, and those with disability reports (*p* < 0.001). In particular, the high chi-square values observed for chronic illness and disability variables suggest that physical health burden is closely associated with depression. Although alcohol use appeared to be lower in the depression group, this finding may be related to reverse causality or health-related restrictions. The markedly higher prevalence of depression among individuals who had experienced spousal loss emphasizes the critical role of social support loss. The higher frequency of depression in obesity and advanced age categories is also consistent with biological vulnerability and functional decline. In contrast, no significant differences were observed for the categories of never married and divorced marital status. Overall, the table strongly demonstrates that depression has multidimensional biopsychosocial determinants (Table 7).

**Table 7:** Categorical variables (Chi-square Test)

Variable	Depression Present *n* (%)	Depression Absent *n* (%)	χ²	*p*	Sig
Gender	2124 (55.6%)	1585 (34.9%)	360.559	< 0.001	***
Living Alone	1159 (30.3%)	968 (21.3%)	89.070	< 0.001	***
Chronic Disease	3277 (85.7%)	3206 (70.5%)	275.637	< 0.001	***
Disability Report	478 (12.5%)	205 (4.5%)	176.246	< 0.001	***
Alcohol Use	405 (10.6%)	810 (17.8%)	86.504	< 0.001	***
Presence of Disability (WG)	2156 (56.4%)	1061 (23.3%)	959.089	< 0.001	***
Marital Status: Never Married	49 (1.3%)	63 (1.4%)	0.098	0.754	
Marital Status: Divorced	137 (3.6%)	157 (3.5%)	0.072	0.789	
Marital Status: Widowed	1844 (48.2%)	1258 (27.7%)	376.441	< 0.001	***
BMI Category: Underweight	71 (1.9%)	39 (0.9%)	15.255	< 0.001	***
BMI Category: Overweight	1504 (39.4%)	2044 (44.9%)	26.361	< 0.001	***
BMI Category: Obese	1070 (28.0%)	1057 (23.2%)	24.521	< 0.001	***
Age 75–84	1281 (33.5%)	1117 (24.6%)	81.055	< 0.001	***
Age 85+	386 (10.1%)	184 (4.0%)	118.972	< 0.001	***

*p < 0.05, **p < 0.01, ***p < 0.001

Depression Present: *n* = 3,822 (45.7%) | Depression Absent: *n* = 4,548 (54.3%)

#### Logistic regression results for all age groups

The overall model results indicate that depression is associated with a multidimensional set of biopsychosocial determinants. The model demonstrated high discriminative ability (10-fold CV AUC = 0.8115), and the McFadden R² value of 0.2446 suggests strong explanatory power within the context of social sciences. The classification performance was consistent with the previous confusion matrix results (Accuracy ≈ 74%; Sensitivity ≈ 66%; Specificity ≈ 81%). Although the Hosmer–Lemeshow test was statistically significant (*p* = 0.001), this finding may be attributable to the large sample size; therefore, model calibration should also be evaluated graphically.

The model results reveal a clear distinction between risk-enhancing and protective factors. Variables associated with an increased risk of depression included female gender (OR = 1.447), poorer perceived health status (OR = 1.49), chronic disease (OR = 1.25), having a disability report (OR = 1.284), and the Washington Group disability indicator (OR = 1.646). These findings suggest that biological vulnerability and physical health burden significantly increase the likelihood of depression. The strongest risk determinant was the happiness variable (OR = 2.516); considering the coding direction, this indicates that lower levels of happiness increase the probability of depression by approximately 2.5 times. This result highlights the strong and direct relationship between subjective well-being and depression.

Protective factors, in contrast, were more closely related to socioeconomic strength and functional independence. Higher educational attainment (OR = 0.914) and increased income (OR = 0.985) were associated with a lower likelihood of depression. Nighttime sleep duration (OR = 0.943), activity status (OR = 0.730), and frequency of physical activity (OR = 0.887) also demonstrated significant protective effects. The most notable protective variables were measures of functional capacity: as Lawton–Brody (OR = 0.386) and Katz (OR = 0.352) scores increased, the likelihood of depression decreased markedly. Overall, this pattern indicates that depression is shaped not only by clinical conditions but also by a holistic risk structure involving functional independence, physical activity, and subjective well-being (Table 8).

**Table 8:** Logistic regression - overall model (Entire Older Population)

Variable	B	SE	Wald z	*p*	OR	95% CI Lower	95% CI Upper	Sig
Constant (Intercept)	-0.9293	0.3218	-2.888	0.004	0.395	0.210	0.742	**
Gender	0.3694	0.0733	5.042	< 0.001	1.447	1.253	1.670	***
Age 75–84	-0.0092	0.0641	-0.144	0.886	0.991	0.874	1.123	
Age 85+	-0.1967	0.1249	-1.575	0.115	0.821	0.643	1.049	
Education Status	-0.0904	0.0195	-4.631	< 0.001	0.914	0.879	0.949	***
Living Alone	0.0597	0.0784	0.762	0.446	1.062	0.910	1.238	
Income (1000 TL)	-0.0155	0.0044	-3.534	< 0.001	0.985	0.976	0.993	***
Health Status	0.3988	0.0433	9.219	< 0.001	1.490	1.369	1.622	***
Chronic Disease	0.2235	0.0720	3.105	0.002	1.250	1.086	1.440	**
Disability Report	0.2501	0.1057	2.365	0.018	1.284	1.044	1.580	*
Alcohol Use	0.0100	0.0825	0.121	0.904	1.010	0.859	1.187	
Night Sleep Duration	-0.0592	0.0170	-3.473	< 0.001	0.943	0.912	0.975	***
Tobacco Use	-0.0193	0.0287	-0.672	0.502	0.981	0.927	1.038	
Activity Status	-0.3152	0.0567	-5.554	< 0.001	0.730	0.653	0.816	***
Frequency of Physical Activity	-0.1196	0.0216	-5.540	< 0.001	0.887	0.850	0.926	***
Happiness	0.9228	0.0410	22.524	< 0.001	2.516	2.322	2.727	***
Presence of Disability (WG)	0.4985	0.0609	8.186	< 0.001	1.646	1.461	1.855	***
Lawton–Brody Total Score	-0.9516	0.1342	-7.093	< 0.001	0.386	0.297	0.502	***
Katz Total Score	-1.0430	0.2086	-5.000	< 0.001	0.352	0.234	0.530	***
Marital Status: Never Married	0.0312	0.2326	0.134	0.893	1.032	0.654	1.627	
Marital Status: Divorced	0.1500	0.1550	0.968	0.333	1.162	0.857	1.574	
Marital Status: Widowed	0.0954	0.0858	1.111	0.266	1.100	0.930	1.302	
BMI Category: Underweight	0.2638	0.2443	1.080	0.280	1.302	0.807	2.101	
BMI Category: Overweight	-0.0371	0.0628	-0.591	0.554	0.964	0.852	1.090	
BMI Category: Obese	-0.0633	0.0728	-0.869	0.385	0.939	0.814	1.083	

Dependent Variable: DEPRESSION_BINARY | Method: Enter | *n* = 8,370

#### Logistic regression results for the 65–74 age group

Table 9 presents the results of the multiple logistic regression analysis identifying factors associated with depressive symptoms among individuals aged 65–74 years (*n* = 5,402). The model explained the presence of depression (*n* = 2,155, 39.9%) at a statistically significant level (McFadden R² = 0.224, AUC = 0.795).

According to the analysis, gender was significantly associated with depression risk (OR = 1.472, 95% CI: 1.233–1.756, *p* < 0.001). Male gender was found to increase the risk of depression by approximately 47% compared with females. Educational level (OR = 0.900, 95% CI: 0.859–0.943, *p* < 0.001) and income level (OR = 0.985, 95% CI: 0.974–0.996, *p* = 0.005) were identified as protective factors, with each increase in educational level reducing the risk of depression by approximately 10%.

When health-related variables were examined, poorer perceived health status (OR = 1.429, 95% CI: 1.284–1.589, *p* < 0.001), the presence of chronic disease (OR = 1.217, 95% CI: 1.025–1.445, *p* = 0.025), and having a disability report (OR = 1.324, 95% CI: 1.018–1.723, *p* = 0.036) were identified as factors increasing the risk of depression.

In terms of lifestyle factors, being active (OR = 0.729, 95% CI: 0.638–0.833, *p* < 0.001), engaging in regular physical activity (OR = 0.917, 95% CI: 0.871–0.965, *p* < 0.001), and obtaining sufficient nighttime sleep (OR = 0.928, 95% CI: 0.888–0.969, *p* < 0.001) showed protective effects. Most notably, perceived happiness emerged as the strongest risk factor (OR = 2.554, 95% CI: 2.315–2.816, *p* < 0.001), which may indicate either reverse coding of the happiness scale or a strong association between unhappiness and depression.

In the assessment of functional capacity, the Lawton–Brody Instrumental Activities of Daily Living (IADL) score (OR = 0.345, 95% CI: 0.239–0.497, *p* < 0.001) and the Katz Activities of Daily Living (ADL) score (OR = 0.443, 95% CI: 0.222–0.882, *p* = 0.020) emerged as strong protective factors. The presence of the Washington Group disability indicator increased the risk by 77% (OR = 1.773, 95% CI: 1.523–2.064, *p* < 0.001). Marital status and BMI categories were not found to be significant in this age group. The model demonstrated acceptable predictive performance, with 10-fold cross-validation yielding 74.7% accuracy, 57.7% sensitivity, and 86.1% specificity.

**Table 9:** Logistic regression results for the 65–74 age group

Variable	B	SE	Wald z	*p*	OR	95% CI Lower	95% CI Upper	Sig
Constant (Intercept)	-0.9374	0.4529	-2.070	0.038	0.392	0.161	0.951	*
Gender	0.3864	0.0903	4.280	< 0.001	1.472	1.233	1.756	***
Education Status	-0.1051	0.0237	-4.430	< 0.001	0.900	0.859	0.943	***
Living Alone	0.0207	0.1052	0.197	0.844	1.021	0.831	1.255	
Income (1000 TL)	-0.0152	0.0055	-2.782	0.005	0.985	0.974	0.996	**
Health Status	0.3567	0.0544	6.557	< 0.001	1.429	1.284	1.589	***
Chronic Disease	0.1963	0.0876	2.240	0.025	1.217	1.025	1.445	*
Disability Report	0.2810	0.1343	2.093	0.036	1.324	1.018	1.723	*
Alcohol Use	0.0240	0.0959	0.250	0.803	1.024	0.849	1.236	
Night Sleep Duration	-0.0751	0.0223	-3.374	< 0.001	0.928	0.888	0.969	***
Tobacco Use	-0.0296	0.0329	-0.900	0.368	0.971	0.910	1.036	
Activity Status	-0.3161	0.0678	-4.659	< 0.001	0.729	0.638	0.833	***
Frequency of Physical Activity	-0.0866	0.0262	-3.301	< 0.001	0.917	0.871	0.965	***
Happiness	0.9375	0.0500	18.764	< 0.001	2.554	2.315	2.816	***
Presence of Disability (WG)	0.5726	0.0777	7.372	< 0.001	1.773	1.523	2.064	***
Lawton–Brody Total Score	-1.0644	0.1864	-5.711	< 0.001	0.345	0.239	0.497	***
Katz Total Score	-0.8147	0.3515	-2.318	0.020	0.443	0.222	0.882	*
Marital Status: Never Married	-0.0999	0.2774	-0.360	0.719	0.905	0.525	1.559	
Marital Status: Divorced	0.1705	0.1794	0.950	0.342	1.186	0.834	1.686	
Marital Status: Widowed	0.1933	0.1092	1.770	0.077	1.213	0.979	1.503	
BMI Category: Underweight	0.0784	0.3561	0.220	0.826	1.082	0.538	2.174	
BMI Category: Overweight	0.0231	0.0798	0.290	0.772	1.023	0.875	1.197	
BMI Category: Obese	0.0029	0.0908	0.032	0.974	1.003	0.839	1.198	

*n* = 5,402 | Depression Present = 2,155 (39.9%) | Depression Absent = 3,247 (60.1%)

#### Logistic regression results for the 75–84 age group

Table 10 presents the factors associated with depressive symptoms among individuals aged 75–84 years (*n* = 2,398). In this age group, the prevalence of depression increased to 53.4% (*n* = 1,281), and the model demonstrated strong explanatory power (McFadden R² = 0.244, AUC = 0.807).

The effect of gender persisted in this age group as well (OR = 1.457, 95% CI: 1.101–1.928, *p* = 0.008). However, notably, the protective effect of educational level lost its statistical significance in this group (*p* = 0.152). Although the protective effect of income level remained, its effect size increased (OR = 0.973, 95% CI: 0.955–0.990, *p* = 0.002).

Among the health-related variables, perceived health status emerged as the strongest risk factor (OR = 1.583, 95% CI: 1.355–1.851, *p* < 0.001). The effect of chronic disease also became more pronounced in this age group (OR = 1.389, 95% CI: 1.049–1.840, *p* = 0.022). In contrast, the presence of a disability report was no longer statistically significant (*p* = 0.411).

With regard to lifestyle factors, activity status (OR = 0.761, 95% CI: 0.611–0.948, *p* = 0.015) and frequency of physical activity (OR = 0.844, 95% CI: 0.778–0.916, *p* < 0.001) continued to exert protective effects, whereas the effect of nighttime sleep became non-significant. Perceived happiness remained a strong risk factor (OR = 2.485, 95% CI: 2.125–2.906, *p* < 0.001).

Indicators of functional capacity gained critical importance in this age group. The protective effect of the Katz Activities of Daily Living (ADL) score became markedly stronger (OR = 0.229, 95% CI: 0.116–0.451, *p* < 0.001), indicating that each one-point increase reduced the risk of depression by 77%. The effect of the Lawton–Brody Instrumental Activities of Daily Living (IADL) score also remained significant (OR = 0.521, 95% CI: 0.337–0.807, *p* = 0.004). The Washington Group disability indicator remained a significant risk factor (OR = 1.511, 95% CI: 1.221–1.870, *p* < 0.001). The Hosmer–Lemeshow test indicated excellent model fit (χ² = 3.47, *p* = 0.901). Overall, the model demonstrated balanced predictive performance (sensitivity: 75.6%; specificity: 70.6%).

**Table 10:** Logistic regression results for the 75–84 age group

Variable	B	SE	Wald z	*p*	OR	95% CI Lower	95% CI Upper	Sig
Constant (Intercept)	-0.8726	0.5866	-1.488	0.137	0.418	0.132	1.319	
Gender	0.3765	0.1429	2.634	0.008	1.457	1.101	1.928	**
Education Status	-0.0557	0.0389	-1.432	0.152	0.946	0.877	1.021	
Living Alone	0.0964	0.1335	0.722	0.470	1.101	0.848	1.431	
Income (1000 TL)	-0.0278	0.0092	-3.025	0.002	0.973	0.955	0.990	**
Health Status	0.4595	0.0796	5.771	< 0.001	1.583	1.355	1.851	***
Chronic Disease	0.3289	0.1433	2.296	0.022	1.389	1.049	1.840	*
Disability Report	0.1619	0.1969	0.822	0.411	1.176	0.799	1.730	
Alcohol Use	-0.1016	0.1753	-0.579	0.562	0.903	0.641	1.274	
Night Sleep Duration	-0.0415	0.0297	-1.401	0.161	0.959	0.905	1.017	
Tobacco Use	-0.0285	0.0642	-0.444	0.657	0.972	0.857	1.102	
Activity Status	-0.2733	0.1120	-2.440	0.015	0.761	0.611	0.948	*
Frequency of Physical Activity	-0.1695	0.0415	-4.085	< 0.001	0.844	0.778	0.916	***
Happiness	0.9103	0.0799	11.391	< 0.001	2.485	2.125	2.906	***
Presence of Disability (WG)	0.4126	0.1088	3.791	< 0.001	1.511	1.221	1.870	***
Lawton–Brody Total Score	-0.6511	0.2230	-2.919	0.004	0.521	0.337	0.807	**
Katz Total Score	-1.4740	0.3455	-4.267	< 0.001	0.229	0.116	0.451	***
Marital Status: Never Married	0.6600	0.4751	1.389	0.165	1.935	0.762	4.910	
Marital Status: Divorced	0.0698	0.3551	0.197	0.844	1.072	0.535	2.151	
Marital Status: Widowed	-0.0001	0.1567	-0.001	0.999	1.000	0.735	1.359	
BMI Category: Underweight	0.1046	0.3908	0.268	0.789	1.110	0.516	2.388	
BMI Category: Overweight	-0.1101	0.1143	-0.964	0.335	0.896	0.716	1.121	
BMI Category: Obese	-0.0861	0.1368	-0.629	0.529	0.918	0.702	1.200	

*n* = 2,398 | Depression Present = 1,281 (53.4%) | Depression Absent = 1,117 (46.6%)

#### Logistic regression results for the 85 + age group

Table 11 presents the factors associated with depressive symptoms among the oldest-old individuals aged 85 years and above (*n* = 570). In this group, the prevalence of depression reached 67.7% (*n* = 386), and the model demonstrated the highest explanatory power (McFadden R² = 0.271).

As a notable finding, gender, which was a strong risk factor in the younger-old age groups, lost its statistical significance in the 85 + age group (OR = 1.270, *p* = 0.415). Similarly, educational level (*p* = 0.635) and income level (*p* = 0.560) were not significantly associated with depression in this age group. This finding suggests that the influence of sociodemographic factors on depression diminishes in advanced old age.

Among the health-related variables, only perceived health status remained a significant risk factor (OR = 1.640, 95% CI: 1.151–2.337, *p* = 0.006). The effects of chronic disease (*p* = 0.713) and disability report (*p* = 0.440) were no longer significant.

With regard to lifestyle factors, frequency of physical activity emerged as the most consistent protective factor (OR = 0.735, 95% CI: 0.603–0.896, *p* = 0.002). Activity status was found to be marginally significant (OR = 0.549, *p* = 0.060). Perceived happiness remained a strong risk factor (OR = 2.429, 95% CI: 1.702–3.465, *p* < 0.001).

An important shift was observed in the functional capacity indicators. The Lawton–Brody Instrumental Activities of Daily Living (IADL) score became the strongest protective factor (OR = 0.210, 95% CI: 0.082–0.537, *p* = 0.001), indicating that each one-point increase reduced the risk of depression by 79%. However, the Katz Activities of Daily Living (ADL) score lost its statistical significance in this group (*p* = 0.105).

As a finding specific to the 85 + age group, obesity was identified as a protective factor (OR = 0.530, 95% CI: 0.295–0.952, *p* = 0.034). This paradoxical result supports the phenomenon referred to as the “obesity paradox” in advanced age and is consistent with findings in the literature suggesting that low BMI in older adults is associated with poorer prognosis. The model demonstrated high sensitivity (84.9%) but low specificity (50.0%), which may be explained by the small sample size.

**Table 11:** Logistic regression results for the 85 + age group

Variable	B	SE	Wald z	*p*	OR	95% CI Lower	95% CI Upper	Sig
Constant (Intercept)	-1.2859	1.2932	-0.994	0.320	0.276	0.022	3.486	
Gender	0.2392	0.2933	0.816	0.415	1.270	0.715	2.257	
Education Status	-0.0427	0.0899	-0.475	0.635	0.958	0.803	1.143	
Living Alone	0.2345	0.2745	0.854	0.393	1.264	0.738	2.165	
Income (1000 TL)	0.0077	0.0132	0.583	0.560	1.008	0.982	1.034	
Health Status	0.4948	0.1806	2.740	0.006	1.640	1.151	2.337	**
Chronic Disease	0.1141	0.3099	0.368	0.713	1.121	0.611	2.058	
Disability Report	0.2913	0.3769	0.773	0.440	1.338	0.639	2.801	
Alcohol Use	0.5887	0.4963	1.186	0.236	1.802	0.681	4.766	
Night Sleep Duration	-0.0038	0.0663	-0.058	0.954	0.996	0.875	1.134	
Tobacco Use	0.1588	0.1901	0.835	0.404	1.172	0.808	1.701	
Activity Status	-0.5993	0.3190	-1.878	0.060	0.549	0.294	1.026	
Frequency of Physical Activity	-0.3078	0.1013	-3.040	0.002	0.735	0.603	0.896	**
Happiness	0.8874	0.1813	4.895	< 0.001	2.429	1.702	3.465	***
Presence of Disability (WG)	0.1287	0.2604	0.494	0.621	1.137	0.683	1.895	
Lawton–Brody Total Score	-1.5618	0.4795	-3.257	0.001	0.210	0.082	0.537	**
Katz Total Score	-0.7409	0.4572	-1.620	0.105	0.477	0.195	1.168	
Marital Status: Never Married	-2.0839	1.6559	-1.258	0.208	0.124	0.005	3.195	
Marital Status: Divorced	0.7317	0.9654	0.758	0.448	2.079	0.313	13.790	
Marital Status: Widowed	-0.2829	0.3510	-0.806	0.420	0.754	0.379	1.499	
BMI Category: Underweight	1.5057	0.8498	1.772	0.076	4.507	0.852	23.840	
BMI Category: Overweight	-0.2213	0.2503	-0.884	0.377	0.801	0.491	1.309	
BMI Category: Obese	-0.6354	0.2989	-2.126	0.034	0.530	0.295	0.952	*

*n* = 570 | Depression Present = 386 (67.7%) | Depression Absent = 184 (32.3%)

#### Comparison across all age groups

Table 12 presents a comparative analysis of the logistic regression results across the three age groups. This comparison is of critical importance for understanding how risk and protective factors for depression change throughout the aging process.

Four factors were found to be consistently significant across all age groups. Perceived health status was a strong risk factor in all groups, and its effect size increased with age (OR: 1.429 → 1.583 → 1.640). Frequency of physical activity was a protective factor in all groups, with its effect size increasing markedly with age (OR: 0.917 → 0.844 → 0.735). Perceived happiness was the strongest risk factor in all groups and remained relatively stable across age groups (OR: 2.554 → 2.485 → 2.429). The Lawton–Brody Instrumental Activities of Daily Living (IADL) score was a strong protective factor in all groups, and its effect increased dramatically with age (OR: 0.345 → 0.521 → 0.210).

The factors whose effects diminished with age were as follows: the effect of gender was significant in the 65–74 (OR = 1.472, *p* < 0.001) and 75–84 (OR = 1.457, *p* = 0.008) age groups, but became non-significant in the 85 + group (*p* = 0.415). Educational level showed a protective effect only in the 65–74 age group (OR = 0.900, *p* < 0.001). Income level was protective in the 65–74 and 75–84 age groups, but lost its effect in the 85 + group. The Washington Group disability indicator was a strong risk factor in the younger-old groups (OR: 1.773 and 1.511), but became non-significant in advanced old age.

The factors that gained importance with age were as follows: the protective effect of the Katz Activities of Daily Living (ADL) score was strongest in the 75–84 age group (OR = 0.229, *p* < 0.001). The BMI-obesity category emerged as a protective factor only in the 85 + age group (OR = 0.530, *p* = 0.034).

**Table 12:** Comparison across age groups

Variable	65–74 OR	65–74 *p*	75–84 OR	75–84 *p*	85 + OR	85 + *p*	Status
Gender	1.472	< 0.001	1.457	0.008	1.270	0.415	Significant only in: 65–74, 75–84
Education Status	0.900	< 0.001	0.946	0.152	0.958	0.635	Significant only in: 65–74
Living Alone	1.021	0.844	1.101	0.470	1.264	0.393	Not significant
Income (1000 TL)	0.985	0.005	0.973	0.002	1.008	0.560	Significant only in: 65–74, 75–84
Health Status	1.429	< 0.001	1.583	< 0.001	1.640	0.006	✓ Significant in all groups
Chronic Disease	1.217	0.025	1.389	0.022	1.121	0.713	Significant only in: 65–74, 75–84
Disability Report	1.324	0.036	1.176	0.411	1.338	0.440	Significant only in: 65–74
Alcohol Use	1.024	0.803	0.903	0.562	1.802	0.236	Not significant
Night Sleep Duration	0.928	< 0.001	0.959	0.161	0.996	0.954	Significant only in: 65–74
Tobacco Use	0.971	0.368	0.972	0.657	1.172	0.404	Not significant
Activity Status	0.729	< 0.001	0.761	0.015	0.549	0.060	Significant only in: 65–74, 75–84
Frequency of Physical Activity	0.917	< 0.001	0.844	< 0.001	0.735	0.002	✓ Significant in all groups
Happiness	2.554	< 0.001	2.485	< 0.001	2.429	< 0.001	✓ Significant in all groups
Presence of Disability (WG)	1.773	< 0.001	1.511	< 0.001	1.137	0.621	Significant only in: 65–74, 75–84
Lawton–Brody Total Score	0.345	< 0.001	0.521	0.004	0.210	0.001	✓ Significant in all groups
Katz Total Score	0.443	0.020	0.229	< 0.001	0.477	0.105	Significant only in: 65–74, 75–84
Marital Status: Never Married	0.905	0.719	1.935	0.165	0.124	0.208	Not significant
Marital Status: Divorced	1.186	0.342	1.072	0.844	2.079	0.448	Not significant
Marital Status: Widowed	1.213	0.077	1.000	0.999	0.754	0.420	Not significant
BMI Category: Underweight	1.082	0.826	1.110	0.789	4.507	0.076	Not significant
BMI Category: Overweight	1.023	0.772	0.896	0.335	0.801	0.377	Not significant
BMI Category: Obese	1.003	0.974	0.918	0.529	0.530	0.034	Significant only in: 85+

### Phase 2: Findings from data-driven analysis

#### Data-driven technique

In this study, Depression_Binary was considered the target variable for predictive modeling. Machine learning models were applied separately to three age categories derived from the new dataset: Under_75, Age_75_84, and Age_85Plus. This approach enabled a refined comparison of predictive performance across age groups.

For each group, a variety of machine learning models were implemented to assess their predictive performance. The models applied in this study included Logistic Regression, Support Vector Machine with RBF kernel (SVC-RBF), Gradient Boosting, AdaBoost, Random Forest, Extra Trees, and K-Nearest Neighbors (KNN). Unlike the previous version of the analysis, model performance was not evaluated solely by accuracy. Instead, accuracy, recall, F1-score, and ROC-AUC were used together to evaluate and compare the performance of these models across different age groups.

To obtain more robust and generalizable results, Stratified K-Fold cross-validation was employed instead of a single 80/20 train-test split. This approach ensured that class proportions were preserved across folds and reduced the likelihood that model performance was affected by a single random partition of the data. In this way, the predictive performance of each algorithm was assessed more rigorously across the different age groups.

To gain deeper insights into the factors influencing depression predictions, feature importance analysis was performed. The models chosen for feature importance assessment were Random Forest and AdaBoost, as these ensemble methods provide robust feature selection capabilities. Feature importance refers to the contribution of each variable to the model’s predictions. In tree-based models such as Random Forest and AdaBoost, feature importance is computed based on how much each feature reduces impurity in the decision trees. A feature that frequently appears in splits and significantly improves node purity is considered more important. This method allows the ranking of features in terms of their influence on model predictions.

Additionally, Logistic Regression was analyzed using feature coefficients, which offer an alternative way of interpreting feature significance. Unlike feature importance, which is based on decision tree-based methodologies [28], feature coefficients measure the direct log-odds relationship between a feature and the target variable [29, 30, 37]. In logistic regression, a positive coefficient indicates that an increase in the feature value is associated with a higher probability of the target outcome (depression), whereas a negative coefficient suggests a lower probability.

There are key differences between feature importance and feature coefficients. Feature importance in tree-based models provides a relative ranking of features but does not directly indicate the direction of the effect, while feature coefficients in logistic regression directly indicate the direction (positive or negative) and magnitude of influence on the target variable [41]. Feature importance is derived from how often a feature is used in splitting nodes in decision trees and the corresponding reduction in impurity, whereas feature coefficients are obtained from maximum likelihood estimation (MLE) in logistic regression and represent how the log-odds of the target variable change with a one-unit increase in the feature. Additionally, feature importance is primarily used in tree-based ensemble models such as Random Forest and AdaBoost, which effectively handle non-linearity and interactions, while feature coefficients are applicable in logistic regression, which assumes a linear relationship between features and the target variable [42, 43].

By employing both feature importance analysis in tree-based models and feature coefficient analysis in logistic regression, this study ensures a comprehensive understanding of the key predictors of depression across age groups. These methods help identify the most influential variables and their relationships with depression, thereby contributing to the development of targeted intervention strategies for different age categories.

Across the age-specific models, predictive performance varied by subgroup, indicating that the most appropriate machine learning algorithm may differ according to age category. In the Under_75 group, Logistic Regression achieved the strongest overall classification performance. In the Age_85Plus group, Random Forest yielded the highest F1-score, suggesting that tree-based ensemble methods may better capture complex predictive structures in the oldest age group. Overall, these findings indicate that depression prediction may require subgroup-specific modeling approaches rather than a single pooled model for all older individuals.

Feature importance analyses based on Random Forest and AdaBoost identified the most influential predictors within each age group, while Logistic Regression coefficients provided additional insight into the direction and magnitude of these associations. Taken together, these findings suggest that the determinants of depression may differ across age-specific subpopulations and that combining multiple machine learning interpretation strategies offers a more comprehensive framework for understanding depression risk in later life.

#### Machine learning-based prediction of geriatric depression across all age groups

##### Machine learning-based prediction of geriatric depression across all age groups

The machine learning models were evaluated for their predictive performance in classifying geriatric depression across all age groups, using multiple metrics beyond simple accuracy (Table 13). Among the evaluated models, Support Vector Machine (RBF) achieved the highest accuracy (0.744), while Gradient Boosting yielded the highest AUC (0.813**)**, indicating the strongest overall discrimination ability. In contrast, Logistic Regression produced the highest recall (0.708) and F1-score (0.715**)**, suggesting that it provided the most balanced performance in identifying depressed individuals while maintaining overall classification stability. These findings indicate that no single model dominated across all performance criteria; rather, different algorithms showed relative strengths depending on the metric considered.

HistGradientBoostin**g** also demonstrated competitive predictive performance, with an accuracy of 0.738, recall of 0.653, F1-score of 0.695, and AUC of 0.805, placing it among the stronger models in the comparison. Random Forest likewise showed solid classification ability **(**accuracy = 0.733, recall = 0.657, F1 = 0.692, AUC = 0.800), although its performance was slightly lower than that of the top-performing models. Extra Trees achieved moderate but acceptable results **(**accuracy = 0.722, F1 = 0.681, AUC = 0.785), supporting its utility as a secondary ensemble-based classifier.

By contrast, K-Nearest Neighbors was the weakest-performing model among those evaluated, with the lowest accuracy (0.695), recall (0.606), F1-score (0.645), and AUC (0.741). Overall, the findings suggest that Logistic Regression, Gradient Boosting, Support Vector Machine (RBF), and HistGradientBoosting provided the most reliable classification performance across all age groups, whereas simpler distance-based methods such as K-Nearest Neighbors were less effective. Taken together, these results highlight the value of comparing multiple complementary evaluation metrics, as the best-performing model may vary depending on whether emphasis is placed on recall, classification balance, or discrimination ability (Table 13).

**Table 13:** Model performance comparison

Model	Accuracy	Recall	F1-score	AUC
Logistic Regression	0.742	0.708	0.715	0.811
Gradient Boosting	0.743	0.654	0.699	0.813
Support Vector Machine (RBF)	0.744	0.679	0.708	0.809
HistGradientBoosting	0.738	0.653	0.695	0.805
Random Forest	0.733	0.657	0.692	0.800
Extra Trees	0.722	0.650	0.681	0.785
K-Nearest Neighbors	0.695	0.606	0.645	0.741

Although Gradient Boosting demonstrated the strongest overall discrimination performance in our predictive modeling, we used permutation importance to interpret the contribution of predictors in the best-performing all-group model. Permutation importance provides a robust and model-agnostic way of evaluating feature relevance by measuring the decline in model performance when the values of a given predictor are randomly shuffled. In contrast to impurity-based importance, permutation importance is less biased toward variables with many categories or continuous ranges and offers a clearer understanding of how strongly each variable contributes to prediction accuracy. For these reasons, permutation importance was preferred to ensure a more interpretable, robust, and comprehensive understanding of the key predictors in the Gradient Boosting model [52, 53].

**Fig. 2:**
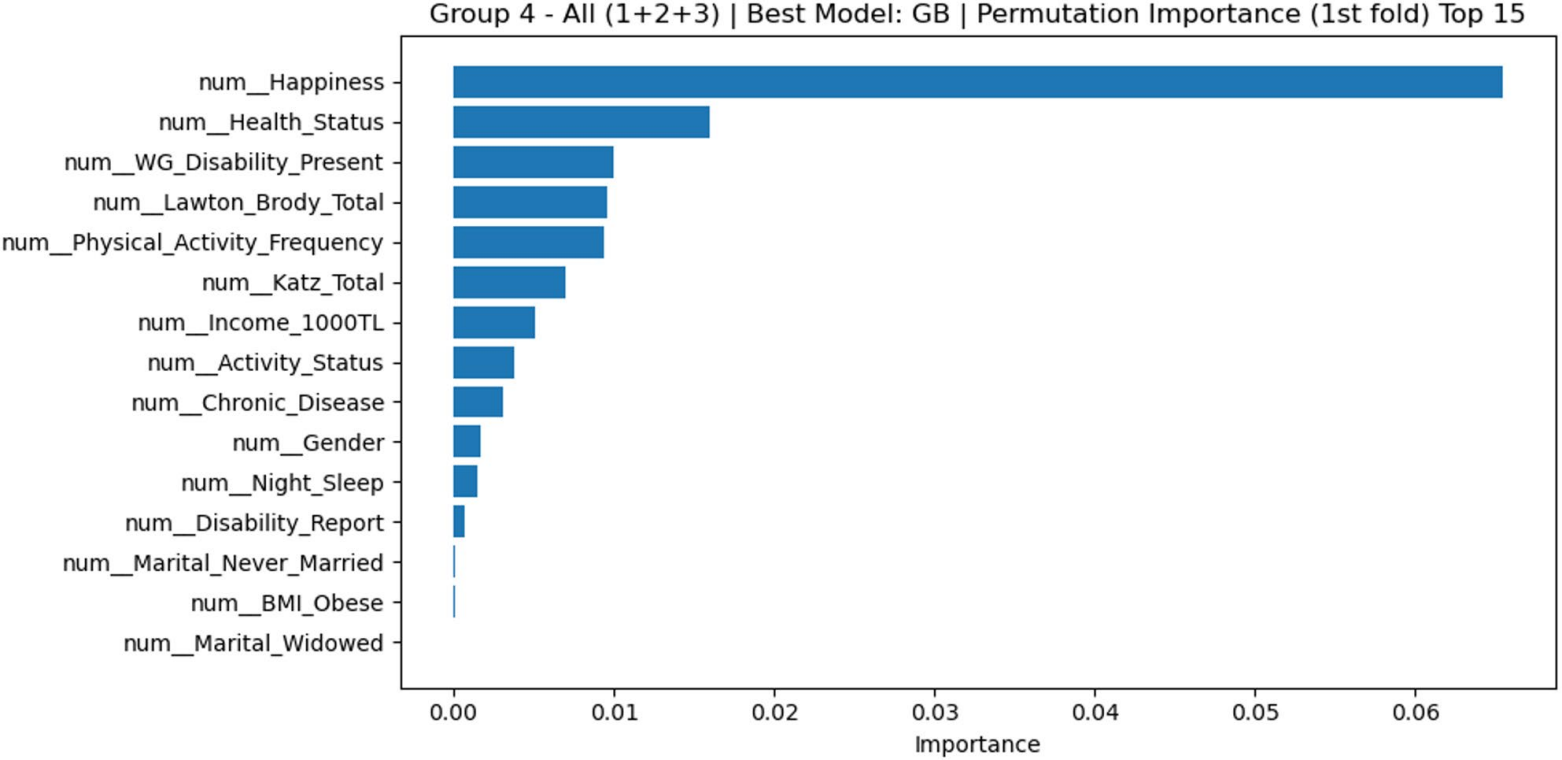
Random forest feature importance and logistic regression feature coefficient

To further investigate the key contributing factors to geriatric depression predictions, feature importance analysis was performed using Gradient Boosting permutation importance, while feature coefficient analysis was conducted for Logistic Regression. The first figure presents the permutation importance rankings from Gradient Boosting, illustrating the most influential predictors identified by the best-performing tree-based ensemble model. Features with higher importance scores indicate variables that played a more substantial role in the prediction of depression. The second figure visualizes feature coefficients derived from Logistic Regression, highlighting both positive and negative associations with depression risk (Fig. 2).

The results from the permutation importance analysis indicate that, in the Gradient Boostin**g** model, the top five most influential predictors were Happiness, Health Status, Disability Status, Lawton–Brody Total Score, and Physical Activity Frequen**cy**. These findings suggest that emotional well-being, perceived health, disability burden, functional independence, and lifestyle-related activity patterns all play major roles in depression risk among older adults. Additional influential predictors included KATZ Total Score, Income Level, Activity Status, Chronic Disease, and Gender, further indicating that geriatric depression is shaped by a multidimensional combination of psychological, physical, and socioeconomic factors.

Furthermore, in the Logistic Regression model, which provides insights into the direction and magnitude of feature influence [39, 54], the most important predictors were identified through their coefficients. Unlike permutation importance in tree-based models, logistic regression coefficients indicate how much a given feature increases or decreases the likelihood of depression. A positive coefficient suggests that an increase in that variable is associated with a higher probability of depression, whereas a negative coefficient implies a protective effect.

The comparison of feature importance across models provides insight into the most relevant predictors of geriatric depression. While Gradient Boosting captures complex non-linear relationships and interaction structures among predictors, Logistic Regression offers a more interpretable framework by explicitly indicating the direction of association. These complementary analyses improve the understanding of depression risk factors and support the development of more targeted interventions for older adults.

In addition, we estimated a global model including age as a continuous predictor. The overall pattern of predictive performance and the identified key variables were broadly consistent with the subgroup analyses, suggesting that the observed associations were not solely an artifact of age categorization but instead reflected meaningful age-related patterns across the aging spectrum.

##### Machine learning-based prediction of geriatric depression in the under 75 group

The machine learning models were evaluated for their predictive performance in classifying depression in the Under_75 group using multiple performance metrics, including accuracy, recall, F1-score, and AUC (Table 14). Among the evaluated models, Gradient Boosting achieved the highest accuracy (0.747) and AUC (0.798), indicating the strongest overall discrimination ability in this age group. However, Logistic Regression yielded the highest recall (0.695) and F1-score (0.675**)**, suggesting that it provided the most balanced performance in identifying depressed individuals while maintaining classification stability. Similarly, Support Vector Machine (RBF) demonstrated competitive performance, with an accuracy of **0.738**, recall of 0.658, F1-score of 0.667, and AUC of 0.794, placing it among the strongest alternative classifiers in this subgroup.

Other ensemble-based methods also showed acceptable predictive ability. HistGradientBoosting achieved an accuracy of 0.737, recall of 0.566, F1-score of 0.632, and AUC of 0.783, while Random Forest produced comparable results (accuracy = 0.735, recall = 0.567, F1 = 0.630, AUC = 0.784). Extra Trees demonstrated moderate performance (accuracy = 0.721, recall = 0.565, F1 = 0.618, AUC = 0.765), whereas K-Nearest Neighbors was the weakest-performing model, with the lowest values across all evaluation metrics (accuracy = 0.695, recall = 0.507, F1 = 0.570, AUC = 0.718). Overall, these findings indicate that Gradient Boosting, Logistic Regression, and Support Vector Machine (RBF) provided the most reliable classification performance for the Under_75 group, while simpler distance-based methods performed less effectively.

To further investigate the factors contributing to depression prediction in the Under_75 group, feature importance analysis was performed using Gradient Boosting permutation importance, as Gradient Boosting was the best-performing model in terms of overall discrimination ability. The permutation importance results showed that the top five most influential predictors were Happiness, Health Status, WG Disability Present, Income Level, and Lawton–Brody Total Score. These findings suggest that emotional well-being, perceived health, disability burden, socioeconomic resources, and functional independence were central determinants of depression risk in individuals under 75 years of age.

Additional important predictors included Education Status, KATZ Total Score, Night Sleep, Physical Activity Frequency, and Activity Status, indicating that depression risk in this group is shaped by a multidimensional combination of psychological, physical, behavioral, and social factors. Lower-ranking but still contributory variables included Gender, Chronic Disease, Tobacco Use, and Disability Report. Overall, the feature importance pattern suggests that depression in the Under_75 group is strongly associated not only with emotional distress but also with broader indicators of health, disability, daily functioning, and lifestyle (Table 14).

**Table 14:** Model Performance comparison for the under_75 group

Model	Accuracy	Recall	F1-score	AUC
Gradient Boosting	0.747	0.568	0.642	0.798
Logistic Regression	0.733	0.695	0.675	0.795
Support Vector Machine (RBF)	0.738	0.658	0.667	0.794
Random Forest	0.735	0.567	0.630	0.784
HistGradientBoosting	0.737	0.566	0.632	0.783
Extra Trees	0.721	0.565	0.618	0.765
K-Nearest Neighbors	0.695	0.507	0.570	0.718

**Fig. 3:**
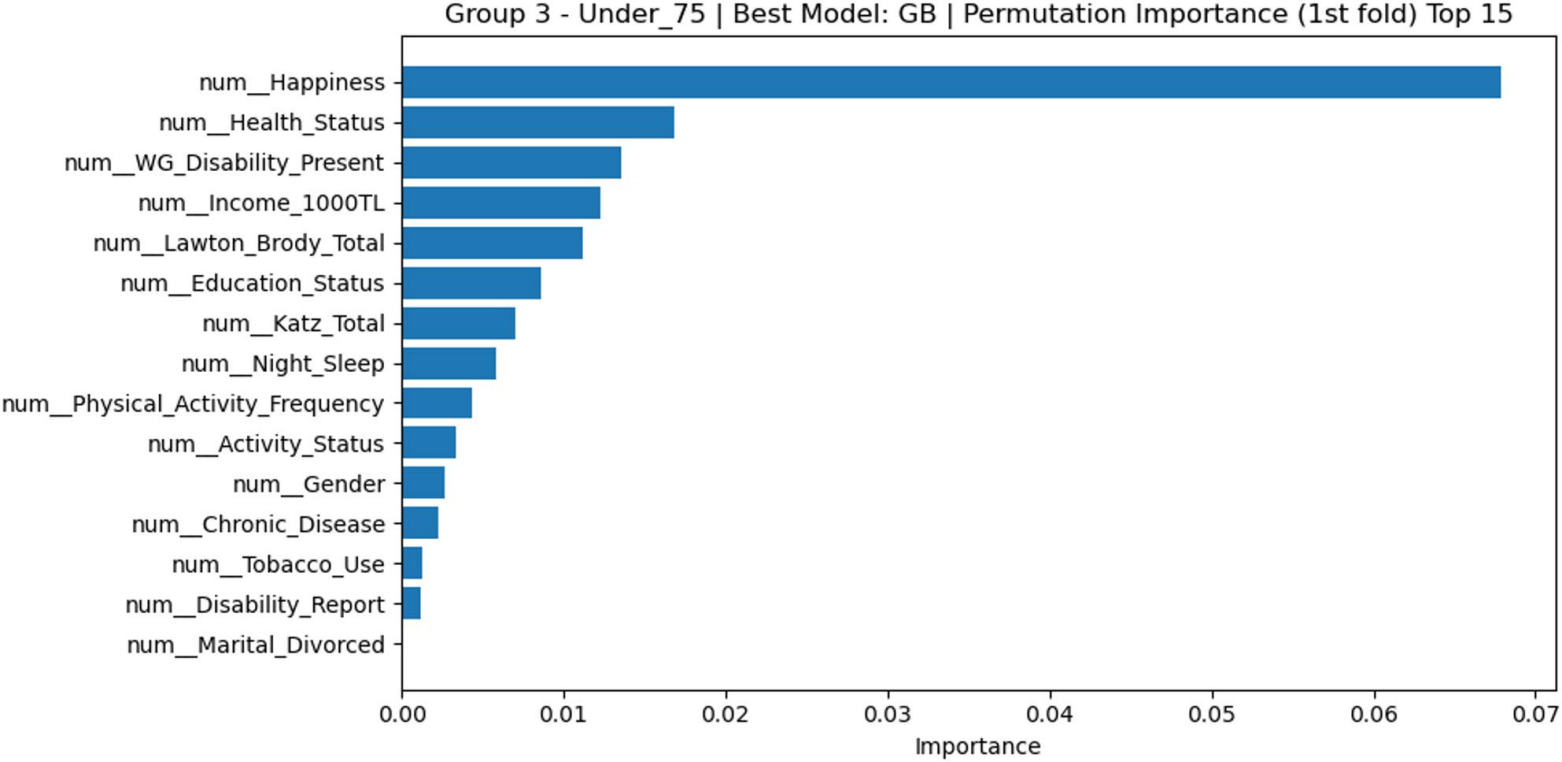
Random forest feature importance and logistic regression feature coefficient

To further investigate the factors contributing to depression prediction in the Under_75 group, feature importance analysis was performed using Gradient Boosting permutation importance, as Gradient Boosting was the best-performing model in terms of overall discrimination ability. The permutation importance results showed that the top five most influential predictors were Happiness, Health Status, WG Disability Present, Income Level, and Lawton–Brody Total Score. These findings suggest that emotional well-being, perceived health, disability burden, socioeconomic resources, and functional independence were central determinants of depression risk in individuals under 75 years of age (Fig. 3).

Additional important predictors included Education Status, KATZ Total Score, Night Sleep, Physical Activity Frequency, and Activity Status, indicating that depression risk in this group is shaped by a multidimensional combination of psychological, physical, behavioral, and social factors. Lower-ranking but still contributory variables included Gender, Chronic Disease, Tobacco Use, Disability Report, and Marital Status (Divorced). Overall, the feature importance pattern suggests that depression in the Under_75 group is strongly associated not only with emotional distress but also with broader indicators of health, disability, daily functioning, lifestyle, and selected sociodemographic characteristics.

##### Machine learning-based prediction of geriatric depression for the 75–84 years age group

The machine learning models were evaluated for their predictive performance in classifying depression in the Age_75_84 group using multiple performance metrics, including accuracy, recall, F1-score, and AUC (Table 15). Among the evaluated models, Support Vector Machine (RBF) achieved the highest AUC (0.807), indicating the strongest overall discrimination ability in this age group. However, Random Forest yielded the highest recall (0.743) **and** F1-score (0.741), suggesting that it provided the most effective balance in identifying depressed individuals while maintaining classification performance. In contrast, Logistic Regression achieved the highest accuracy (0.729), demonstrating that linear modeling also performed competitively in this subgroup.

Gradient Boosting also showed strong predictive ability, with an accuracy of 0.723, recall of 0.741, F1-score of 0.740, and AUC of 0.801, placing it very close to the best-performing models overall. Similarly, HistGradientBoosting and Extra Trees produced moderate but acceptable classification performance, with F1-scores of 0.728 and 0.728, respectively. In contrast, K-Nearest Neighbors was the weakest-performing model in this subgroup, with the lowest values across all evaluation metrics (accuracy = 0.673, recall = 0.672, F1 = 0.687, AUC = 0.723). Overall, these findings indicate that Support Vector Machine (RBF), Random Forest, Gradient Boosting, and Logistic Regression provided the most reliable classification performance for the Age_75_84 group, whereas simpler distance-based methods were less effective.

To further investigate the factors contributing to depression prediction in the Age_75_84 group, feature importance analysis was performed using permutation importance based on the Support Vector Machine (RBF) model, as this model achieved the highest AUC and was therefore identified as the best-performing classifier in terms of discrimination ability. The permutation importance results showed that the top five most influential predictors were Happiness, Health Status, Lawton–Brody Total Score, WG Disability Present, and Income Leve**l**. These findings suggest that emotional well-being, perceived health, functional independence, disability burden, and socioeconomic resources were central determinants of depression risk in individuals aged 75–84 years.

Additional influential predictors included KATZ Total Score, Physical Activity Frequency, Gender, Marital Status (Never Married), and Education Status, indicating that depression risk in this age group is shaped by a multidimensional combination of psychological, physical, functional, behavioral, and sociodemographic factors. Lower-ranking but still contributory variables included Night Sleep, Chronic Disease, Marital Status (Widowed), Disability Report, and Activity Status. Overall, the feature importance pattern suggests that depression in the Age_75_84 group is strongly associated not only with emotional distress and general health perception but also with daily functioning, disability, economic status, and selected social background characteristics (Table 15).

**Table 15:** Model performance comparison (75–84 years)

Model	Accuracy	Recall	F1-score	AUC
Support Vector Machine (RBF)	0.723	0.699	0.729	0.807
Logistic Regression	0.729	0.713	0.737	0.805
Gradient Boosting	0.723	0.741	0.740	0.801
Random Forest	0.722	0.743	0.741	0.792
HistGradientBoosting	0.710	0.726	0.728	0.780
Extra Trees	0.711	0.728	0.728	0.774
K-Nearest Neighbors	0.673	0.672	0.687	0.723

To further investigate the factors contributing to depression prediction in the Age_75_84 group, feature importance analysis was performed using permutation importance based on the Support Vector Machine (RBF) model, as this model achieved the highest AUC and was therefore identified as the best-performing classifier in terms of discrimination ability. The permutation importance results showed that the top five most influential predictors were Happiness, Health Status, Lawton–Brody Total Score, WG Disability Present, and Income Level. These findings suggest that emotional well-being, perceived health, functional independence, disability burden, and socioeconomic resources were central determinants of depression risk in individuals aged 75–84 years.

**Fig. 4:**
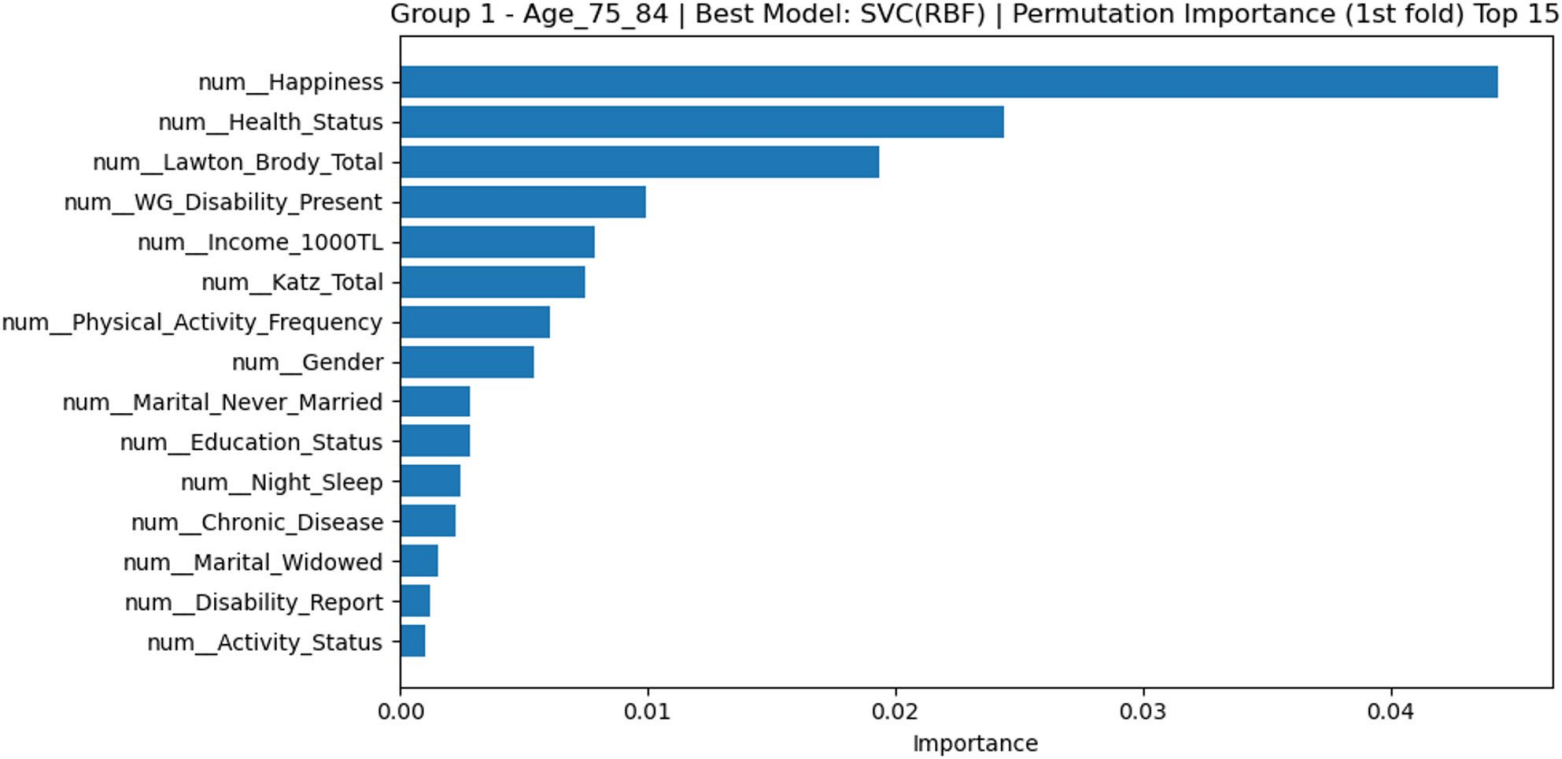
Random forest feature importance and logistic regression feature coefficient

Additional influential predictors included KATZ Total Score, Physical Activity Frequency, Gender, Marital Status (Never Married), Education Status, Night Sleep, and Chronic Disease. Importantly, the model also assigned smaller but still measurable contributions to Marital Status (Widowed), Disability Report, and Activity Status, indicating that these factors also participated in the prediction of depression, even though their relative importance was lower than that of the leading variables. Overall, the feature importance pattern suggests that depression in the Age_75_84 group is shaped not only by emotional distress and general health perception but also by daily functioning, disability, socioeconomic conditions, sleep, and selected marital and activity-related characteristics (Fig. 4).

##### Machine learning-based prediction of geriatric depression for the ≥ 85 years age group: model performance and key predictors

The machine learning models were evaluated for their predictive performance in classifying depression in the Age_85Plus group using multiple performance metrics, including accuracy, recall, F1-score, and AUC (Table 16). Among the evaluated models, Support Vector Machine (RBF) achieved the highest AUC (0.795), indicating the strongest overall discrimination ability in this age group. However, Gradient Boostin**g** yielded the highest accuracy (0.735**)**, while Random Forest achieved the highest recall (0.850**)**. In terms of balanced classification performance, Random Fores**t and** Gradient Boosting produced the strongest F1-scores (0.810 each), suggesting that these ensemble-based methods provided the most effective overall performance in identifying depressed individuals in the oldest age group.

HistGradientBoosting and Extra Trees also demonstrated strong predictive performance, with F1-scores of 0.805 and 0.803, respectively, supported by high recall values (0.834 and 0.837). Logistic Regression remained competitive, with an accuracy of 0.726, recall of 0.738, F1-score of 0.785, and AUC of 0.784, indicating that linear modeling still captured meaningful depressive risk patterns in this subgroup. In contrast, K-Nearest Neighbors showed the weakest discrimination ability, with the lowest AUC (0.713), although its recall remained relatively high (0.842). Overall, these findings indicate that Random Forest, Gradient Boosting, HistGradientBoosting, Extra Trees, and Support Vector Machine (RBF) provided the most reliable classification performance for the Age_85Plus group, whereas simpler distance-based models were less stable in terms of overall discrimination.

To further investigate the factors contributing to depression prediction in the Age_85Plus group, feature importance analysis was performed using permutation importance based on the Support Vector Machine (RBF) model, as this model achieved the highest AUC and was therefore identified as the best-performing classifier in terms of discrimination ability. The permutation importance results showed that the top five most influential predictors were Happiness, Lawton–Brody Total Score, KATZ Total Score, Physical Activity Frequency, and Activity Status. These findings suggest that emotional well-being, functional independence, daily living capacity, and activity-related behaviors were central determinants of depression risk among individuals aged 85 years and older.

Additional influential predictors included Health Status, BMI Overweight, WG Disability Present, BMI Underweight, and Gender, indicating that depression risk in this age group is shaped not only by psychological and functional factors but also by nutritional status, disability burden, and demographic characteristics. Lower-ranking but still contributory variables included Education Status, BMI Obese, Night Sleep, Income Level, and Marital Status (Divorced). Overall, the feature importance pattern suggests that depression in the Age_85Plus group is strongly associated with emotional distress and reduced functional capacity, while body composition, physical activity, disability, and selected sociodemographic characteristics also contribute meaningfully to prediction (Table 16).

**Table 16:** Model performance comparison (≥ 85 years age group)

Model	Accuracy	Recall	F1-score	AUC
Support Vector Machine (RBF)	0.711	0.705	0.766	0.795
Random Forest	0.730	0.850	0.810	0.792
Logistic Regression	0.726	0.738	0.785	0.784
Extra Trees	0.723	0.837	0.803	0.775
Gradient Boosting	0.735	0.832	0.810	0.774
HistGradientBoosting	0.726	0.834	0.805	0.771
K-Nearest Neighbors	0.702	0.842	0.792	0.713

To further investigate the factors contributing to depression prediction in the Age_85Plus group, feature importance analysis was performed using permutation importance based on the Support Vector Machine (RBF) model, as this model achieved the highest AUC and was therefore identified as the best-performing classifier in terms of discrimination ability. The permutation importance results showed that the top five most influential predictors were Happiness, Lawton–Brody Total Score, KATZ Total Score, Physical Activity Frequency, and Activity Status. These findings suggest that emotional well-being, functional independence, daily living capacity, and activity-related behaviors were central determinants of depression risk among individuals aged 85 years and older.

**Fig. 5:**
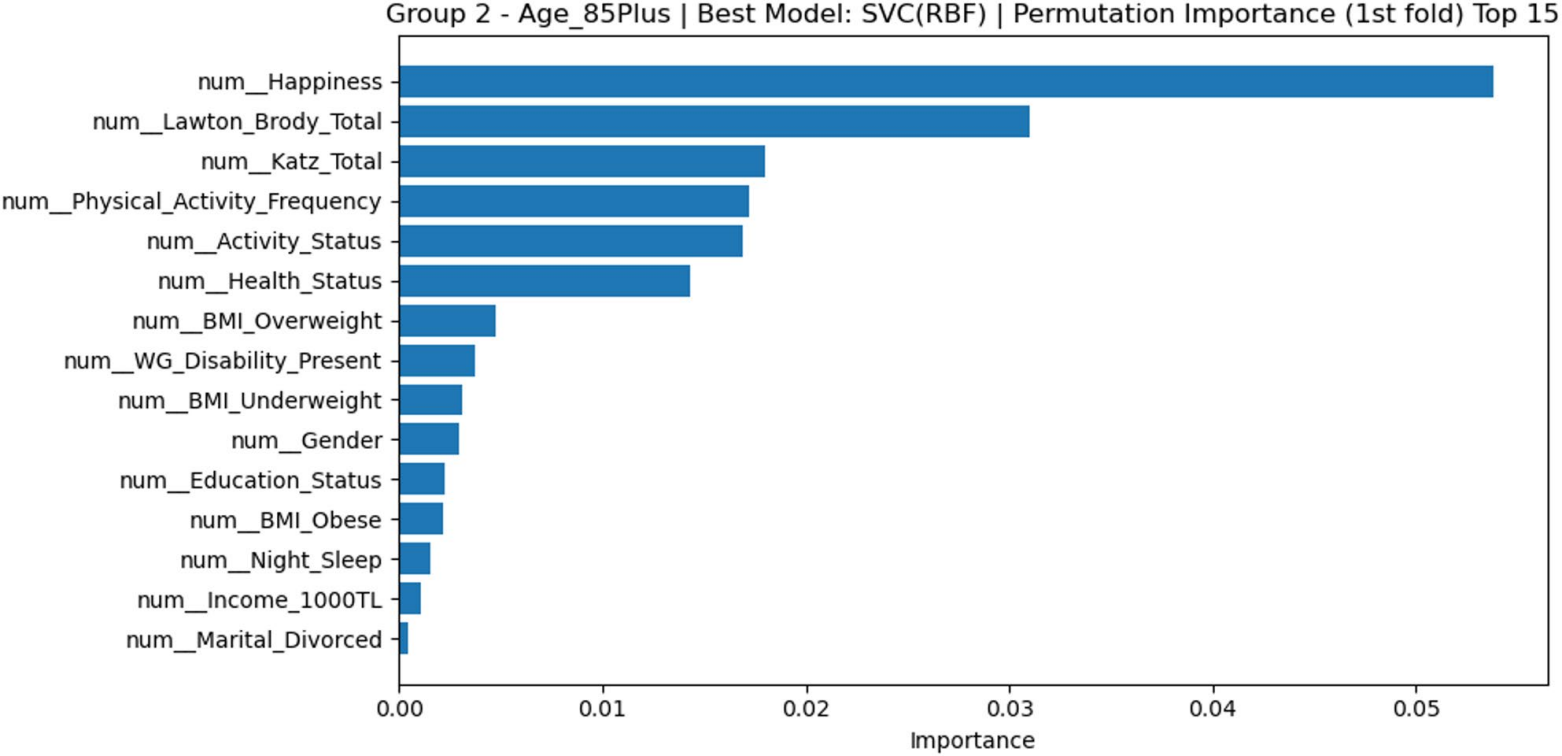
Random forest feature importance and logistic regression feature coefficient

Additional influential predictors included Health Status, BMI Overweight, WG Disability Present, BMI Underweight, and Gender, indicating that depression risk in this age group is shaped not only by psychological and functional factors but also by nutritional status, disability burden, and demographic characteristics. Importantly, the model also assigned smaller but still measurable contributions to Education Status, BMI Obese, Night Sleep, Income Level, **and** Marital Status (Divorced), showing that these variables also participated in the prediction of depression, even though their relative importance was lower than that of the leading predictors. Overall, the feature importance pattern suggests that depression in the Age_85Plus group is strongly associated with emotional distress and reduced functional capacity, while body composition, physical activity, disability, socioeconomic conditions, and selected sociodemographic characteristics also contribute meaningfully to prediction (Fig. 5).

## Discussion

### Summary of principal findings

In this study, risk factors for geriatric depression among individuals aged 65 years and older were examined using both theory-driven logistic regression (LR) and data-driven machine learning (ML) approaches, based on a nationally representative dataset (TYPA-2023, *n* = 8,370). The overall LR model, which included 24 independent variables, explained approximately one-quarter of the variance in depression (McFadden R² = 0.245) and demonstrated good discriminative performance in 10-fold cross-validation (AUC = 0.812). Among the seven ML algorithms evaluated on the full sample, Gradient Boosting achieved the highest AUC (0.813), while Logistic Regression yielded the best recall (0.708) and F1-score (0.715), confirming its utility as a balanced and interpretable classifier. No single model dominated across all performance criteria; ensemble methods (Gradient Boosting, Random Forest, HistGradientBoosting) and Support Vector Machine (RBF) provided competitive discrimination, whereas simpler distance-based approaches (K-Nearest Neighbors) performed less well.

Across both analytical frameworks, psychosocial distress (unhappiness level), self-rated health status, functional independence (Lawton–Brody IADL), and frequency of physical activity emerged as the most consistently important predictors. Age-stratified analyses revealed meaningful heterogeneity: in the young-old (65–74), psychosocial and socioeconomic factors predominated, whereas in the oldest-old (≥ 85), physical health deterioration, functional dependency, and body composition gained prominence. These patterns were robust across analytic approaches, supporting the study’s a priori hypothesis that the balance of depression determinants shifts from psychosocial to physical/functional domains with advancing age.

### Cross-age consistency: universal predictors of geriatric depression

A central finding of this study is that four predictors maintained statistical significance across all three age strata (65–74, 75–84, ≥ 85) in the logistic regression models, constituting a “core axis” of geriatric depression risk. These same variables also ranked consistently among the top features in the permutation importance profiles of the best-performing ML models—Gradient Boosting for the overall and Under_75 samples, and Support Vector Machine (RBF) for the 75–84 and 85 + samples—providing cross-method convergence that strengthens the credibility of the findings.

***Unhappiness level*** emerged as the strongest risk factor across all models and age groups (overall LR model OR = 2.516; age-specific ORs: 2.554 in 65–74, 2.485 in 75–84, 2.429 in ≥ 85). Each one-unit deterioration on the happiness scale increased the odds of depression by approximately 2.4–2.6 times. The modest downward gradient from younger-old to oldest-old suggests that emotional well-being remains a universally powerful predictor, though its relative magnitude shows slight attenuation in very advanced age where physical and functional determinants gain prominence. In the overall Gradient Boosting model, happiness ranked first in permutation importance, confirming its dominance across analytical approaches. Consistent with prior geriatric mental health literature, the association between negative affect and depression is well established; the contribution of the present study lies in demonstrating how this association’s magnitude varies by late-life stage within the same nationally representative dataset and harmonized analytic framework.

***Self-rated health status*** was a strong and consistent risk factor in all groups, with an effect size that increased with age (OR: 1.429 → 1.583 → 1.640). This trajectory indicates that subjective health perception becomes an increasingly powerful correlate of depression as individuals age. In the oldest-old, where multimorbidity is nearly universal, the individual’s subjective evaluation appears to capture a dimension of vulnerability that objective clinical indicators alone may not fully represent. Health status also ranked second in the overall Gradient Boosting permutation importance, reinforcing the LR finding.

***Functional independence (Lawton–Brody IADL)*** was a consistently significant protective factor, and its effect size increased dramatically with age (OR: 0.345 → 0.521 → 0.210). In the 85 + group, each unit increase in Lawton–Brody score was associated with approximately a 79% reduction in depression odds—the strongest protective effect observed in the study. The Lawton–Brody total score also ranked among the top five features in permutation importance across all age-specific ML models. This finding supports the established view that decline in instrumental activities of daily living plays a critical role in the development of late-life depression, and extends it by showing that the magnitude of this protective effect intensifies with advancing age.

***Frequency of physical activity*** was the fourth universally significant protective factor, with an effect size that also increased with age (OR: 0.917 → 0.844 → 0.735). This suggests that the protective value of regular physical activity strengthens as individuals move into more advanced age categories. Physical activity frequency ranked among the top five permutation importance features in both the overall Gradient Boosting and the 85 + SVM models. This finding has important clinical implications: physical activity interventions may yield even greater benefits for depression prevention among the oldest-old.

The consistency of these four factors across methods and age strata suggests that psychosocial well-being, subjective health perception, functional independence, and physical activity constitute a fundamental cluster of geriatric depression determinants that should form the basis of universal screening and intervention strategies.

### Age-specific determinants: differential risk profiles

Beyond the universally significant factors, several predictors exhibited age-specific significance patterns, supporting the original rationale for age stratification. These differential profiles indicate that late-life stages differ not because of discrete thresholds but because the relative balance between psychosocial, socioeconomic, and physical/functional drivers of depression shifts with age.

***Gender.*** Female gender was associated with a significantly elevated depression risk in the 65–74 (OR = 1.472, *p* < 0.001) and 75–84 (OR = 1.457, *p* = 0.008) age groups, corresponding to a roughly 43–47% higher odds compared with males. However, this effect lost significance in the 85 + group (*p* = 0.415). The attenuation likely reflects convergence in health burden: gender role expectations, caregiving load, and hormonal factors that disproportionately affect younger-old women may become less salient when both sexes face comparable levels of frailty and dependency. This pattern was mirrored in the ML feature importance analyses, where gender ranked higher among predictors in the Under_75 model than in the 85 + model. These findings suggest that gender-focused depression intervention programs may be most effective in the 65–84 age range.

***Education.*** Educational attainment showed a significant protective effect only in the 65–74 age group (OR = 0.900, *p* < 0.001), with diminished and non-significant effects in the 75–84 (*p* = 0.152) and 85+ (*p* = 0.635) groups. In the ML analyses, education ranked among the top predictors in the Under_75 Gradient Boosting model but declined in importance at older ages. The cognitive reserve and health literacy advantages conferred by formal education appear most influential in the earlier phases of late life, while more immediate determinants—health deterioration, functional decline, and loss of autonomy—overshadow educational attainment in advanced old age.

***Income.*** Income was a significant protective factor in the 65–74 (OR = 0.985, *p* = 0.005) and 75–84 (OR = 0.973, *p* = 0.002) groups, with each 1,000 TL increase in monthly income reducing depression risk by 1.5–2.7%. Income lost significance in the 85 + group (*p* = 0.560). Permutation importance corroborated this pattern: income ranked fifth among predictors in the Under_75 model but declined substantially in the 85 + model. Socioeconomic resources appear to exert their strongest influence in younger late life, when financial security facilitates social participation, healthcare access, and coping capacity.

***Chronic disease and disability.*** Chronic disease was a significant risk factor in the 65–74 (OR = 1.217, *p* = 0.025) and 75–84 (OR = 1.389, *p* = 0.022) groups but became non-significant in the 85 + group (*p* = 0.713). The Washington Group disability indicator followed a parallel trajectory: it was a strong risk factor in the younger-old groups (OR: 1.773 and 1.511) but lost significance in advanced old age. This diminishing discriminative power likely reflects the near-universality of chronic illness and disability in the oldest-old, reducing between-individual variability. In the ML analyses, WG disability ranked third in the overall Gradient Boosting model but declined in relative importance in the 85 + model, where functional measures (IADL, ADL) and activity-related variables were more dominant.

***Katz ADL.*** The Katz ADL score showed a significant protective effect in the 65–74 (OR = 0.443, *p* = 0.020) and 75–84 (OR = 0.229, *p* < 0.001) age groups, with the strongest effect in the middle-old—each unit increase was associated with approximately a 77% reduction in depression risk. In the 85 + group, the effect was in the expected direction but did not reach significance (OR = 0.477, *p* = 0.105), likely due to the smaller sample size (*n* = 570). Notably, the ML models compensated for this limitation: Katz ADL ranked among the top five permutation importance features in the 85 + SVM model, demonstrating the complementary value of the hybrid analytic framework in detecting predictive contributions that LR alone may miss due to statistical power constraints.

***The obesity paradox.*** An intriguing age-specific finding was that obesity emerged as a significant protective factor exclusively in the 85 + group (OR = 0.530, *p* = 0.034). This finding supports the “obesity paradox” documented in the geriatric literature, according to which higher BMI in very advanced age may be associated with better survival, nutritional reserve, and functional outcomes. BMI-related categories (overweight and obese) also appeared among the moderately influential predictors in the 85 + ML model, corroborating that body composition plays a differential role in the oldest-old. In this population, preservation of adequate nutritional status and bodily reserves may function as a buffer against depressive symptomatology.

***Sleep and activity level.*** Night sleep duration was protective only in the 65–74 group (OR = 0.928, *p* < 0.001), suggesting that sleep patterns acquire a more complex structure with advancing age. General activity level was significant in the 65–74 and 75–84 groups but not in the 85 + group (*p* = 0.060). However, the ML analyses captured what the LR missed: activity status and physical activity frequency both ranked among the top five permutation importance features in the 85 + model, illustrating how the data-driven approach detected predictive relationships that LR—constrained by reduced sample size—was unable to confirm at conventional significance thresholds.

Taken together, these age-specific patterns demonstrate a clear shift from psychosocial and socioeconomic drivers in the young-old to physical/functional determinants in the oldest-old. The attenuation of predictors such as education, gender, and income in the 85 + group should not be interpreted as absence of relevance but rather as a shift in the balance of determinants as physical and functional constraints intensify with advancing age.

### Comparison with existing literature

The finding that unhappiness/negative affect is the strongest predictor of geriatric depression is consistent with a large body of prior evidence [55–57]. However, the present study extends this literature by quantifying how the magnitude of this association varies across late-life stages within the same nationally representative dataset and harmonized analytic framework. The strong association between self-rated health and depression aligns with established research showing that subjective health perception is a more powerful predictor of mental health outcomes than objective clinical indicators alone [58]. The increasing effect size of self-rated health with advancing age provides new evidence for a stage-dependent strengthening of this association.

The protective role of functional independence (IADL and ADL) is well-documented in the geriatric depression literature [59, 60]. The present findings corroborate and extend this evidence by demonstrating that the protective effect of Lawton–Brody IADL scores increases dramatically with age, reaching its strongest magnitude in the 85 + group. This age-dependent intensification has important implications for clinical practice, suggesting that rehabilitation programs promoting daily living activities [61] may yield the greatest mental health benefits when targeted at the oldest-old population.

The present findings also align with international evidence indicating that depression in later life is strongly shaped by psychosocial vulnerability, social isolation, loneliness, and perceived quality of life. In a systematic review of 53 studies, Silva et al. [62] reported that depression among older adults during the COVID-19 pandemic was associated with multiple psychosocial and contextual determinants, including loneliness, social isolation, reduced social interaction, and vulnerability related to health and living conditions. This broader international evidence supports the interpretation of the present findings, particularly the strong predictive role of unhappiness, poorer self-rated health, functional limitation, and reduced physical activity. Although the present study was not designed as a COVID-19-specific analysis, its results are consistent with the view that late-life depression should be understood as a multidimensional condition shaped by emotional, functional, social, and health-related factors rather than by clinical morbidity alone.

Post-pandemic evidence further suggests that depressive symptoms among older adults may persist beyond the acute phase of COVID-19 and remain closely linked to loneliness and perceived quality of life. In a cross-sectional study conducted in Portugal, Silva et al. [63] found that depressive symptomatology was significantly associated with loneliness and quality of life among both institutionalised and non-institutionalised older adults. This finding is relevant to the present study because the strongest predictors identified in our models—unhappiness, self-rated health, functional independence, disability burden, and physical activity frequency—reflect not only clinical vulnerability but also broader dimensions of subjective well-being and everyday life functioning. Therefore, the current findings from Türkiye add to international evidence by showing that these psychosocial and functional determinants remain important within a nationally representative older population and that their relative importance changes across age strata.

The gender-related findings are consistent with the broader literature documenting higher depression rates among older women [64], while the attenuation of the gender effect in the 85 + group adds nuance. Prior studies have reported mixed results regarding gender differences in very advanced age; the present findings suggest that gender convergence in health burden may underlie this phenomenon. The protective role of socioeconomic factors (education and income) in the younger-old, and their diminishing significance with age, is consistent with prior research indicating that the influence of distal socioeconomic determinants is attenuated in advanced age by more proximal health and functional factors [65]. The obesity paradox observed exclusively in the 85 + group aligns with a growing body of geriatric research suggesting that higher BMI in very advanced age may be associated with survival advantage and better nutritional reserves.

In terms of methodological comparison, the present study’s integration of theory-driven (LR) and data-driven (ML) techniques provides a more comprehensive analytical framework than studies relying on a single approach. The LR–ML convergence on the same core set of determinants substantially strengthens the interpretability of the findings: while LR enhances causality interpretation and effect size analysis [66], ML-based methods reveal predictive patterns that may be obscured by sample size limitations or the linearity assumptions of traditional models [13, 65]. Importantly, the ML models identified predictive contributions from variables that did not reach statistical significance in LR—most notably Katz ADL and activity status in the 85 + group—demonstrating that the combined approach is not merely confirmatory but genuinely additive.

A recent study based on the same nationally representative Türkiye Elderly Profile Survey 2023 further supports the psychosocial interpretation of late-life depression. Firat and Kurutkan examined the determinants of the Perceived Ageism Index using an integrated theory-driven and data-driven framework and reported that geriatric depression was the strongest risk factor across all age groups, both dimensions of perceived ageism, and both analytical methods [67]. Their findings also showed that social participation and digital competence functioned as protective factors against specific dimensions of perceived ageism, while psychosocial vulnerability, social exclusion, digital disadvantage, and healthcare inequities shaped older adults’ perceived ageism experiences. These findings are highly relevant to the present study because they demonstrate, within the same national survey context, that geriatric depression is closely embedded in a broader network of psychosocial disadvantage, social participation, digital inclusion, and perceived discrimination. Therefore, the current depression-focused analysis complements this emerging Turkish evidence by identifying the age-specific determinants of depression itself, while the perceived ageism study highlights how depression may also operate as a central psychosocial vulnerability marker in later life.

Additional international evidence further supports the interpretation of late-life depression as a condition closely embedded in social connectedness, perceived isolation, and psychosocial vulnerability. In a longitudinal mediation analysis of older Americans, Santini et al. showed that social disconnectedness predicted higher perceived isolation, which in turn was associated with subsequent symptoms of depression and anxiety [68]. Similarly, Robb et al., in a large survey of older adults in London during the early phase of the COVID-19 pandemic, reported that social isolation indicators were associated with poorer mental health outcomes, including anxiety and depression [69]. These findings are consistent with the present study, in which unhappiness, poorer self-rated health, functional limitation, disability burden, and reduced physical activity emerged as central correlates of depressive symptoms among older adults.

The broader pandemic-related literature also indicates that loneliness and social isolation became highly prevalent among older adults and may have contributed to persistent mental health vulnerability. Su et al., in a systematic review and meta-analysis including 30 observational studies and 28,050 participants, reported pooled prevalence estimates of 28.6% for loneliness and 31.2% for social isolation among older adults during the COVID-19 pandemic [70]. Furthermore, Choi et al., in a systematic review on social isolation and behavioral health in older adults, concluded that both subjective and objective forms of social isolation are associated with adverse behavioral health outcomes, including depressive symptoms, sleep disturbance, and fatigue [71]. Taken together, these studies strengthen the international relevance of the present findings and support the view that geriatric depression should be interpreted within a multidimensional framework that includes emotional well-being, perceived health, functional independence, social participation, loneliness, and social isolation.

Among the ML algorithms, the finding that no single model dominated across all performance criteria echoes recent evidence in geriatric prediction research. Ensemble methods (Gradient Boosting, Random Forest) excelled in discrimination (AUC), while Logistic Regression offered the best balance of recall and F1-score, confirming its continued utility for interpretable depression risk modeling. The age-specific variation in best-performing algorithms—Logistic Regression in the Under_75 group, Random Forest in the 85 + group—suggests that depression prediction may require subgroup-specific modeling strategies rather than a single pooled model.

### Practical and policy implications

The findings carry several implications for clinical practice and public health policy. The universal significance of unhappiness, self-rated health, functional independence, and physical activity across all age groups supports the development of transdiagnostic screening instruments that incorporate these four dimensions. In primary care settings, a brief assessment combining subjective well-being, self-rated health, and functional status items could serve as an efficient gateway for identifying older adults at elevated depression risk, irrespective of age.

However, the significant age-related heterogeneity observed in this study underscores the necessity of tailoring interventions by life stage. In the 65–74 age group, where education, income, and gender emerge as salient predictors, interventions emphasizing psychosocial support programs [64], health literacy enhancement, and economic security may yield the greatest returns. Gender-focused depression programs—addressing caregiving burden, social isolation, and role transitions—appear most justified in this age range. In the 75–84 age group, the strengthening of functional status indicators (Katz ADL) and the persistence of income effects suggest that integrated models combining socioeconomic support with rehabilitation-oriented care may be optimal.

In the 85 + age group, where physical health, functional independence, and body composition become the dominant determinants, policy emphasis should shift toward rehabilitation programs promoting activities of daily living [61], nutritional monitoring to prevent both malnutrition and unintended weight loss (given the obesity paradox), and physical activity programs adapted to the functional capacity of the oldest-old. The fact that ML models detected predictive contributions from variables that LR could not confirm at conventional significance levels in this group suggests that clinical decision support systems for the oldest-old may benefit from ML-augmented screening tools that capture nonlinear and interaction-based risk patterns.

From a resource allocation perspective, these findings provide an empirical basis for age-stratified intervention design: rather than applying uniform depression prevention protocols, healthcare systems could prioritize socioeconomic and psychosocial supports for the young-old and functional rehabilitation and nutritional care for the oldest-old, thereby maximizing the efficiency of limited public health resources.

### Strengths and limitations

This study has several notable strengths. First, it draws upon a nationally representative dataset (TYPA-2023) covering the entire elderly population of Türkiye (*n* = 8,370), providing broad generalizability within the national context. Second, the hybrid LR–ML analytic framework represents a methodological advance over single-approach studies: the convergence of findings across theory-driven and data-driven methods strengthens the robustness and interpretability of the identified determinants. Third, the age-stratified design with three gerontologically grounded subgroups enabled the detection of differential risk profiles that would have been masked in a global model. Fourth, the use of permutation importance as a model-agnostic feature ranking method provided harmonized comparability across ML algorithms, and multiple sensitivity analyses (alternative GDS cut-offs, ordinal models, continuous-score regressions, age modeled continuously with splines) confirmed the robustness of the findings.

Several limitations must be acknowledged. First, the cross-sectional design precludes causal inference; the observed associations should be interpreted as statistical relationships rather than evidence of causality. Terms such as “protective” or “risk increasing” used throughout this discussion denote the direction of statistical association, not causal mechanisms. Second, 11.2% of the data were excluded using listwise deletion due to missing values, which may have introduced selection bias. Third, the limited sample size of the 85 + group (*n* = 570) reduced statistical power for this subgroup; the non-significance of certain variables (e.g., Katz ADL) in LR may partly reflect power limitations rather than true absence of association, as confirmed by the ML models’ detection of these variables’ predictive contributions.

Fourth, depression was identified using a self-report instrument (GDS-30) rather than a clinical diagnostic interview, which may limit diagnostic accuracy. Although binary dichotomization of GDS scores reflects a screening-oriented use case, it may entail information loss; however, sensitivity analyses with alternative cut-offs, ordinal models, and continuous-score regressions yielded consistent results. Fifth, the study relied on self-reported data for all measures, introducing potential recall bias and social desirability bias. Sixth, some predictors—particularly functional status measures and unhappiness—demonstrated unusually strong associations with depression, raising the possibility of conceptual overlap with depressive symptomatology. Multicollinearity diagnostics confirmed that unhappiness level and Katz ADL were independent (VIFs < 2), whereas Lawton–Brody IADL, health status, BMI, and age exhibited higher collinearity. These variables were retained due to their clinical importance, but findings should be interpreted with this caveat.

Seventh, although age stratification into three categories aligns with gerontological convention and facilitates clinically meaningful subgroup comparisons, it inevitably reduces granularity and may obscure within-group variability. Sensitivity analyses modeling age as a continuous predictor with nonlinear terms yielded largely consistent results, suggesting that the categorical approach enhanced clinical interpretability without altering substantive conclusions. Eighth, because different ML algorithms yield algorithm-specific importance metrics that are not directly comparable, we adopted permutation importance as a model-agnostic harmonization method. While this approach improves cross-model comparability, it does not capture interaction-specific contributions or direction of effect, which were complemented by LR coefficients. Finally, while logistic regression assumes linearity in the log-odds, the complementary ML analyses were specifically designed to detect potential nonlinear and higher-order associations, and class balance (MCC corroborated performance ordering) supported the informative use of accuracy alongside other metrics. Future research should employ longitudinal designs, clinical diagnostic instruments, and composite indices or factor-analytic approaches to mitigate the limitations noted above.

## Conclusion

In this study, the determinants of geriatric depression in individuals aged 65 and above were examined using both traditional logistic regression and machine learning-based methods. The study utilized a subjective self-reported dataset derived from the Turkish Elderly Profile Survey. In real-world data, the use of subjective assessments reflecting individuals’ mental states is commonly preferred and holds significant importance in clinical applications. This dataset is uniquely valuable as it represents the entire elderly population of a developing country, allowing for a comprehensive, data-driven and theory-driven analysis of the factors influencing depression burden among older adults in Turkey. By providing empirical evidence on the early diagnosis and intervention of depression in later life, this study plays a fundamental role in the development of public health policies.

The findings reveal that psychosocial factors (particularly unhappiness level), health status, daily living activities, and socioeconomic indicators are key determinants of depression risk. Across all age groups, unhappiness level was found to increase depression risk by approximately 2.4 to 2.6 times (OR range: 2.429–2.554), while poor health status and greater dependency levels contributed to a higher risk of depression. Furthermore, among machine learning models, ensemble methods (AdaBoost, Gradient Boosting, and Bagging Classifier), as well as Logistic Regression, demonstrated superior performance in both accuracy and interpretability.

Subgroup analyses by age categories revealed that education and socioeconomic factors played a more dominant role in the 65–74 age group, whereas physical health and functional impairments were more critical in the 85 + age group. These findings contribute significantly to both the academic literature and policy-making efforts by enhancing the understanding of the multidimensional nature of geriatric depression and supporting the development of age-specific intervention strategies.

Overall, the findings align with our a priori hypotheses by demonstrating meaningful heterogeneity in depression correlates across the young-old, middle-old, and oldest-old, with a shift from psychosocial to physical/functional drivers in advanced age.

## Data Availability

The data used in this study, titled Age Related Determinants of Geriatric Depression, are part of the Turkish Health Survey, a microdata set provided by the Turkish Statistical Institute (TÜİK). Researchers can access this anonymized dataset upon application to TÜİK and payment of the required fee. Additionally, The dataset used in this study is publicly available and can be accessed through the following links: Google Sheets: [https://docs.google.com/spreadsheets/d/1WxPLRczYtDxm7kGC0c7QVG3JDbFTzqQt/edit?gid=1176251330#gid=1176251330]. Kaggle: [https://www.kaggle.com/datasets/drfatihorhan/age-related-determinants-of-geriatric-depression].
